# The cost of language: functionally over-dominant language circuits in the human brain may limit cognitive abilities and non-verbal executive functions

**DOI:** 10.3389/fnhum.2025.1654703

**Published:** 2025-11-06

**Authors:** Hans-Peter Lipp

**Affiliations:** Institute of Evolutionary Medicine, University of Zurich, Zurich, Switzerland

**Keywords:** evolution, neural networks, motor neocortex, neural attractor, brain development, prodigies, language evolution, social stratification

## Abstract

Evolutionarily, the most recent connective system in the human brain is the language circuitry. However, its presence may impose restrictions on higher executive functions apparent as non-verbal talents in art, science, and management– essentially a conflict between talking and doing. Since the associative cortex underlies thinking, the question then is how much of it is assigned to language functions, and how much is left for associative networks that support non-verbal functions such as planning and parallel processing. Arguments: (i) The determinant of neocortical network organization is the motor cortex, which acts as the main attractor for all processes in the hemispheres yet is split in two sub-attractors formed by disproportionally enlarged zones of origins for two bundles, the corticospinal tract co-driving movements of arms and hands, and the corticobulbar tract to the motor nuclei of the cranial nerves innervating the vocal tract, tongue and face. (ii) This arrangement must entail different functional properties of the associated networks. The language network faces executive limits because the linear generation of words becomes dominated by cerebellar feedback from lingual processing (“one word generates the next”), while the non-verbal networks have more freedom in generating mental goals and movements. (iii) Functional imbalance between these neocortical networks results from altered connections caused by neuronal competition during brain development, either by epigenetic events or by selectable genetic factors. (iv) The descent of the larynx in humans during the paleolithic period and the following self-domestication and neoteny during the last 30,000 years have favored the expansion of the cerebral language network. Voices gained prosody and melody, thereby transmitting fine-grained levels of emotions between individuals, facilitating the evolution of collective cooperation in agricultural economies. On the other hand, with the advent of densely populated kingdom states, emotional voicing also enabled mass control of people for warfare and social stratification of societies. This new environment entailed genetic adaptation of a large population segment resulting in moderately lowered cognition, firstly by expansion of the language network permitting emotional association of simple memes and words, possibly supported by additional mechanisms conserving a child-like stage of brain development responsible for word-linked beliefs.

## Introduction

There is a common belief that the progressing evolution of language ensured social stabilization, facilitated empathy, and mitigated social tensions, thus accounting for the ethical, technical, and scientific development of humanity by transgenerational addition of knowledge. This is not untrue as far as it concerns the bidirectional communication between parents and children, teachers and students, and familiar persons in a limited constellation resembling a hunter-gatherer tribe. However, human language has other properties when it serves as a unidirectional communication tool such as political propaganda, warmongering, marketing, or plain hate speech pervading social networks despite of algorithmic filtering. Likewise, synchronized actions of humans can be triggered easily by absolute key words linked with simple visual memes (Mehravipour, 2025), mostly directing them to collective aggressive behavior, verbal or even physical. Apparently, evolution has primed the human brain to be susceptible to this partially maladaptive yet poorly investigated non-verbal aspect of language. Similarly, such reactivity appears to be linked to a simplistic way of thinking and uncritical beliefs making it possible to manipulate psychologically large segments of the population in modern states by opinion makers, spin doctors or artificial intelligence, thus creating a social stratification between administrative and economical elites dominating and profiting from the so-called common people. At a more sophisticated intellectual level, lengthy discussions about the correct meanings of words and their appropriate usage have spread massively, specifically in WEIRD countries - western, educated, industrialized, rich, democratic (Henrich et al., 2010) suggesting that many educated persons appear to think preferentially in verbal terms. But how to explain such observations obviously linked to brain organization and processing?

The past decades have seen much progress in deciphering the neural organization of language, specifically by imaging. Such activity mapping includes functional magnetic resonance imaging (fMRI) for slow changes depending on blood flow, rapid alterations detected by magnetoencephalography (MEG), and sophisticated imaging of standard electroencephalography (EEG) yet with high-resolution. Likewise, analysis was complemented by digital analysis of static magnetic resonance analysis (MRI) and by the development of large data sets permitting comparisons of results from different laboratories (Mandal et al., 2023). These studies have led to the identification of a rather specific complex neural network devoted to language, as contrasted to a multidomain (MD) and some other networks involved in other aspects of thinking (Fedorenko and Varley, 2016; Fedorenko et al., 2024b). In fact, the language network appears to involve large regions of the associative neocortex (Hertrich et al., 2020) and so the question emerges whether a permanently active language network is interfering with other domains of brain processing supporting higher executive functions apparent as non-verbal talents in art, science, and management– essentially a conflict between talking and doing.

This paper interprets the language network as an evolutionary newcomer that has remodeled, by genetic and epigenetic processes during the last 30,000 years, the pattern of human brain connections resulting in people with different thinking modes, thereby facilitating the emergence of antique and modern mega societies. The aim is to provide a neuroethological, neuroanatomical and evolutionary framework of how language and its cerebral processing activate emotions, synchronize human behavior and prime specific modes of thinking. The paper does not explicitly deal with linguistics but rather focuses on the ethological aspects of language.

The arguments making such a scenario plausible are presented in seven sections followed by a discussion:

1. The connectome of the “old” brain and its functions
2. The motor cortex and its interactions with the associative cortex regions
3. Dividing the associative cortex into audiomotor and visuomotor networks
4. How the two cortical motor output systems may shape different thinking
5. The forebrain as a playground for mammalian evolution
6. From Pleistocene to Logocene – the price of integration
7. Some neurosociology: dimming the developing brain to stabilize modern societies?

## The connectome of the “old” brain and its functions (1)

From a neuroethologist’s point of view, the evolution of the mammalian cortex simply reflects the addition of computational capacity, varying with the ecological requirements of a given species, and being high in humans. Conversely, the structural layout of neuronal groups and fiber connections in basal forebrain and upper brainstem appears rather similar across vertebrate species, as it corresponds to the phylogenetically conserved “minimal brain” or “old brain” able to maintain sufficiently sophisticated motor outputs to ensure survival in various ecological niches. Evolutionarily, the midbrain and the brain stem clearly remain the commanding regions of the mammalian brain, since these brain parts receive all exteroceptive and interoceptive inputs, either directly or indirectly through the basal forebrain system, and can organize the essential motor output according to species. Thus, the mammalian cortex is primarily refining and analyzing subcortical inputs while behavioral output reflects essentially the cortical feedback to the subcortical drivers, as visualized by Supplementary Figure 1 from Lipp and Wolfer (2022). Functionally, the “old brain” is holding a complex repertoire of species-specific motor programs to activate sequences of movements in the spinal cord, for example the locomotor center (Ferreira-Pinto et al., 2018) or separate networks for biting attacks and pursuit locomotion (Han et al., 2017). In fact, it can maintain some cognitive and behavioral functionality after removal of the entire neocortex in dogs and rats (Goltz, 1892; Huston and Borbely, 1974), but also in rodents with developmentally lacking neocortex (Ferris et al., 2019; Turan, 2021).

## The motor cortex and its interactions with the associative cortex regions (2)

The paper presents a “motocentric” perspective: nervous systems of all animal species that move actively have evolved through locomotion and collecting sensory information that guides further movements which then select new information. Ongoing motor activity and its proprioceptive feedback is priming the activity of even sensory brain parts in different species, such as flies (Aimon et al., 2019), mice (Stringer et al., 2019; Parker et al., 2020), nematodes (Kaplan et al., 2020; Kaplan and Zimmer, 2020) and lampreys (Grillner et al., 2008). But the human brain shows some remarkable specializations in terms of organizing its motor output.

### The motor homunculus and its output organization

**FIGURE 1:**
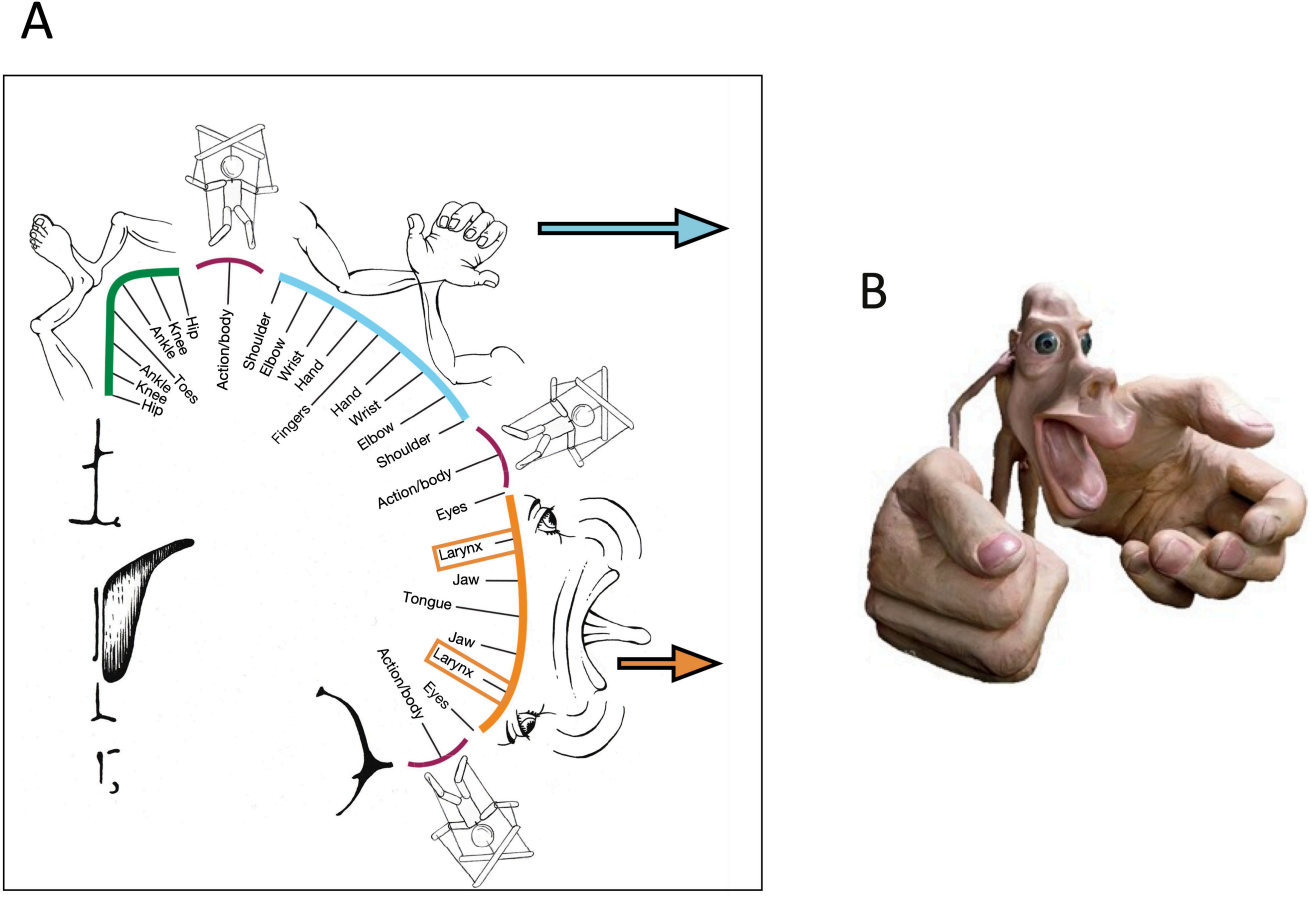
Modern “homunculus” in the motor cortex. **(A)** The scheme maps points from which electrical stimulation elicited muscle specific movements, the puppet player symbols indicate sites from which stimulation elicited coordinated muscular responses. Blue arrow indicates the corticospinal tract, orange the corticobulbar tract. Drawings modified after Gordon et al. (2023) under the Creative Common Attribution License 4.0. **(B)** Figurine exaggerating the relations between manual and linguistic output. Taken and slightly edited from a public blog of the Cabrera laboratory (https://blog.cabreraresearch.org/tag/neuroscience).

Figure 1A presents the classic output system of the human neocortex, as obtained by electrical stimulation of the surface and responses from muscles, in a modernized version. It shows a somatotopic image of the human muscular system, feet, legs, and trunk on top, followed by a disproportionately enlarged region activating muscles of hands and thumb, and more ventrally a similarly enlarged region containing the pyramidal cells sending axons to the muscles of face, tongue, pharynx and larynx. There have been earlier mapping studies but only Penfield and Boldrey (1937) provided drawings showing these relations as a grotesquely shrunken human, “homunculus”, and a modeled figure from Penfield’s laboratory exaggerating these features became famous (Figure 1B). The homunculus concept has been criticized for oversimplicity (Catani, 2017; Saadon-Grosman et al., 2020; Ghimire et al., 2021) but was eventually redefined by showing an intricate internal organization of the primary motor cortex (Gordon et al., 2023; Deo et al., 2024). The modified homunculus now includes three additional zones forming a system for whole-body action planning, intertwined with effector-specific regions where stimulation elicits specific muscle movements. The twin network of the motor homunculus is the sensory homunculus in proximity to the motor cortex, and this strict somatotopic relation is maintained at various levels of the sensorimotor system and the cerebellum (Supplementary Figure 2). However, irrespective of the intrinsic complication of the motor homunculus (Graziano and Aflalo, 2007), it abuts into two final motor output paths.

**FIGURE 2:**
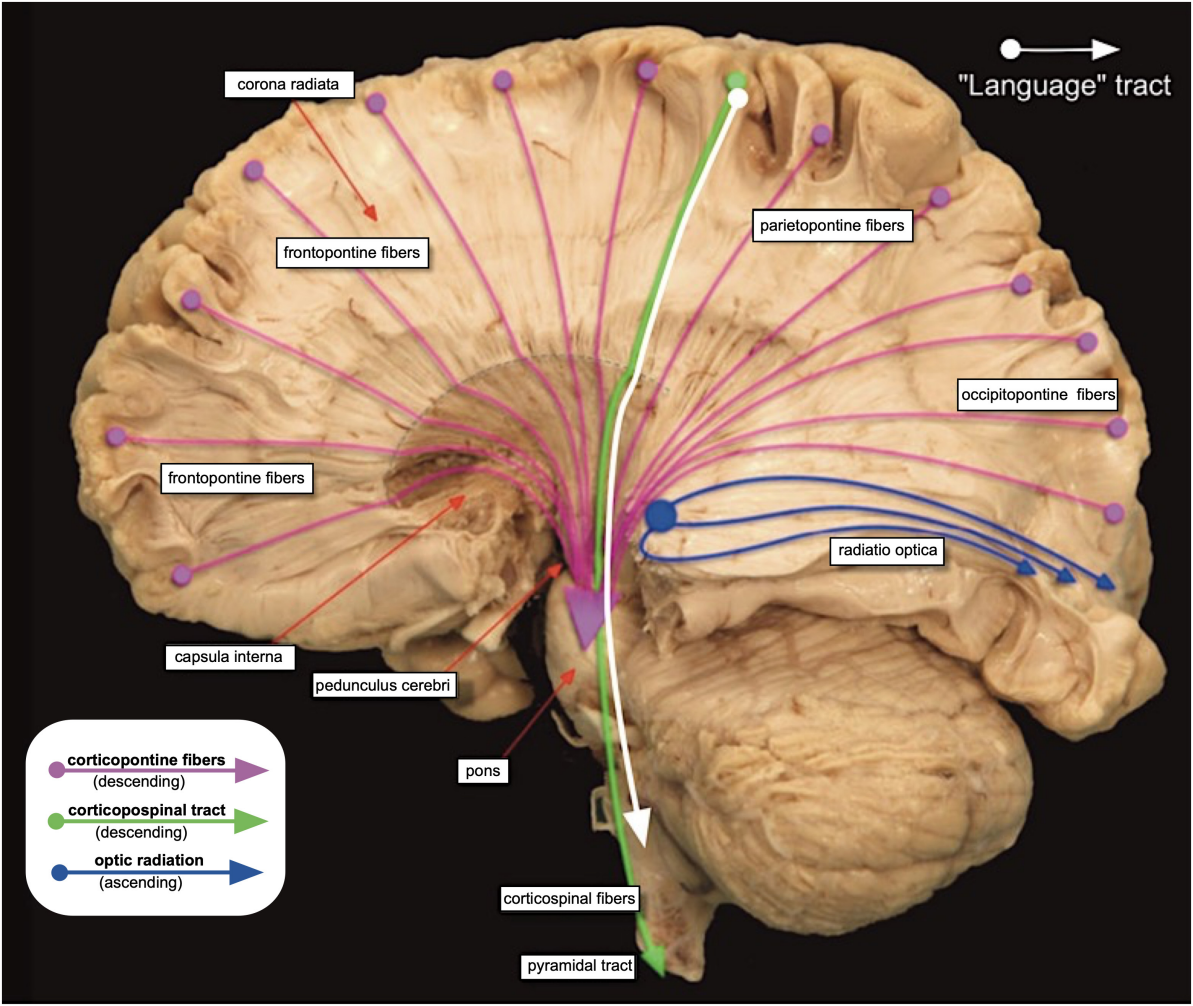
Anatomical preparation of the corona radiata and capsula interna showing the descending outputs of the cerebral cortex. Purple fibers indicate the various fiber tracts to the pontine region (corticopontine tracts) while the green arrow symbolizes the corticospinal and the white arrow the corticobulbar (layrngo-pharyngeal) tract. Note that most descending fibers originate from all associative cortex regions. Note also that the size of the motor bundles is much larger at the cortical origin than drawn here. They dwindle by giving off many collaterals. Image taken and modified with permission from the Department of Neurosciences of the University Medical Center in Amsterdam (https://anatomy-neurosciences.com/education/humanbrain/tracts-human, Image 4).

The output organization is evident from Figure 2 and contains two bundles. One of them [about one million fibers (Tomasch, 1969)] reaches as corticobulbar tract the supraspinal motor networks including the motoneurons of the various nuclei of origin of the cranial nerves, plus some cervical segments of the spinal cord controlling movements of head, neck, and diaphragm. The other bundle, also about one million axons (Tomasch, 1969), continues as the classic corticospinal pyramidal tract to reach the motoneurons in the ventral horn of the spinal cord for fine-tuning the movements of extremities, arms, and hands, while the last fibers taper off to drive movements of body and legs. Like other corticopontine fibers, both tracts give off numerous collaterals to thalamus, basal ganglia, and to diencephalic and mesencephalic nuclei not to be discussed here. Taken together, these two output systems represent only 5%–10% of the descending cortical axons, and in these tiny final bundles the fibers are likely arranged in somatotopic order as imposed by the motor cortex (Karbasforoushan et al., 2022). In other words, the output of the entire forebrain is compressed into two small fiberoptic-like cables carrying a microscopic image of the vocal or manual motor cortex, respectively. Given a very big system with such specific output sinks, one is tempted to see them as organizers or point attractors (Knierim and Zhang, 2012; Khona and Fiete, 2022) or final network hubs to use the connectomist terminology. Taken together, the human neocortex can be considered as a rather simple structure, essentially a living projection screen dynamically scrambling thalamic and other subcortical inputs but eventually fitting its activity pattern into the double somatotopic output matrix of the homunculus.

### The principal role of the associative cortex

**FIGURE 3:**
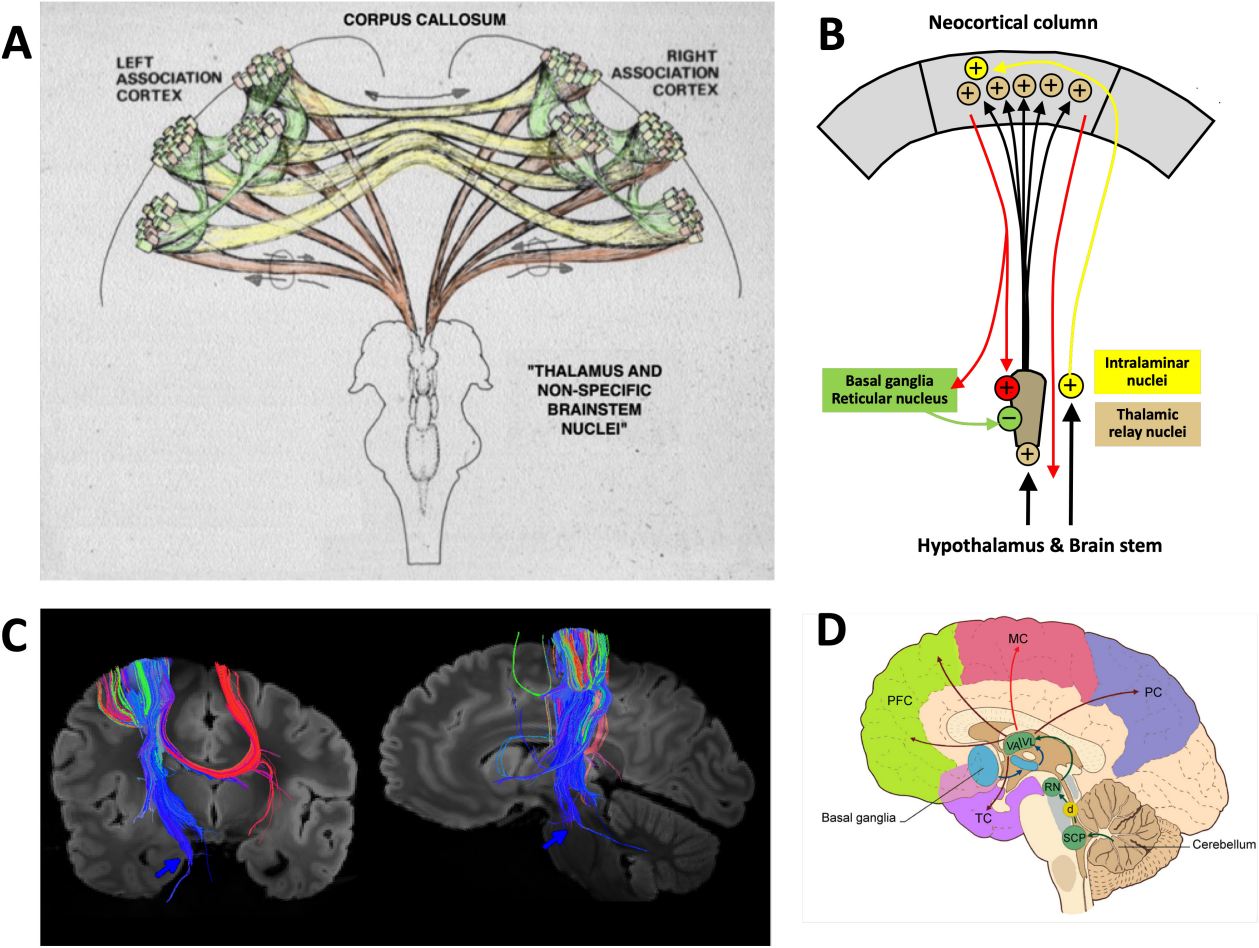
Different views of the associative cortex and its cortical and subcortical network. **(A)** Artistic interpretation of the radial organization of the neocortex as sculpted by ascending fibers from thalamus and upper brainstem. Brown: thalamocortical fibers, green: intracortical network between clusters of cortical columns, yellow: callosal fibers. Drawing modified from Lipp and Wolfer (2022) under the Creative Common Attribution License 4.0. **(B)** Scheme of an associative thalamo-cortical channel and its regulatory loops. **(C)** Tractography in an adult post-mortem brain showing callosal and cortico-pontine fiber tracts. Blue: descending corticopontine tracts with ending regions (arrow) and fibers to the cerebellum (arrow). Red: callosal projections to homotopic and heterotopic areas. Picture taken from Wilkinson et al. (2016) under Creative Commons Attribution License 4.0. More details in Figure 4 of the publication**. (D)** Distribution of thalamo-cortical axons from ventrolateral and anterior thalamus to many associative regions. Note that the cerebellar feedback targets primarily the motor and sensorimotor cortex, but cerebellar fibers diverge into many thalamic nuclei projecting to various cortical association regions. Most likely, the latter nuclei receive also inputs from hypothalamus and other subcortical structures. Taken from Palesi et al. (2015) under Creative Commons Attribution License 4.0.

**FIGURE 4:**
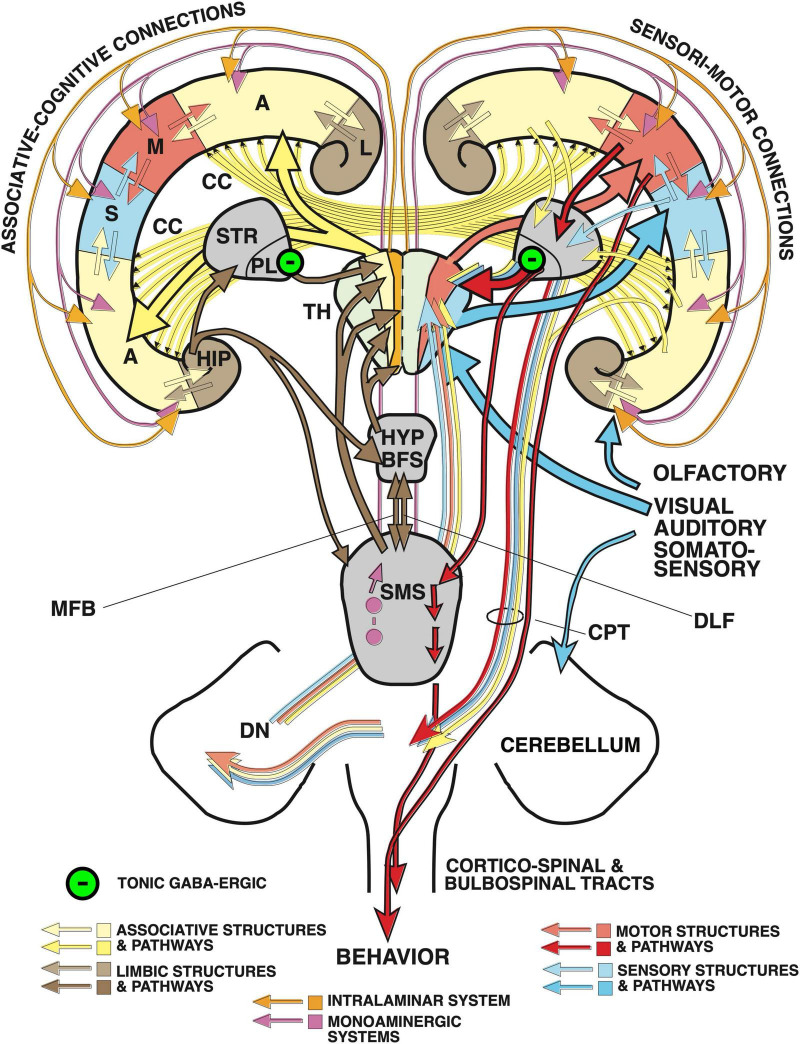
“Cognitive” and “non-cognitive” circuitry of the mammalian brain. At right, the main connections of the primary sensory (S) and motor cortex areas (M). The left side illustrates the connectivity of the “cognitive” (associative and limbic) cortex. Principal inputs to the associative cortex originate in the hypothalamus (HYP) and the reticular formation (RF) and reaches the non-specific (associational) thalamus (TH, yellow) from where ascending divergent fibers reach many parts of the associative cortex (A). The connections of the associative cortex include reciprocal fiber connections with neighboring areas subserving tangential spread of information. Part of the output of the associative cortex is fed into the motor fine-tuning loops, to the other hemisphere via the corpus callosum (CC), and via polysynaptic chains into the (marginal) limbic cortex (L) and the hippocampal formation (HIP). The cingular and entorhinal cortex send also fibers to the limbic basal ganglia (STR) where the limbic pallidum (PL) sends (tonically inhibitory) GABAergic fibers to the intralaminar system of the thalamus and to the anterior thalamic nuclei. Other fibers from hippocampus and amygdala reach the hypothalamus and reticular formation, both structures sending fibers to the associative thalamus from which divergent axons spread again to the associative cortex portions. Thus, the activity of the entire cortex can be structured through ascending control systems: the intralaminar system (orange), and the ascending monoaminergic systems (purple), see also text. Any alteration in these comparatively minor structures, be it genetic, developmental, or pathological, has thus a potentially powerful impact. Figure and legend taken from Lipp and Wolfer (2022) and modified under Creative Commons Attribution License 4.0.

Computationally, the associative cortex provides the fundamental model for neocortical function. To understand verbal and non-verbal functions, one needs to consider first its connective design, specifically its various inputs and their balance. Figures 3, 4 present various perspectives visualizing the relations between subcortical structures and associative neocortex.

The neocortex is traditionally conceived as a horizontal matrix formed by neurons and intersecting axons. However, the patterning of neuronal activity within the neocortical network is primarily radially organized by the ascending axons from the thalamus (Goldman-Rakic, 1987; LaMantia and Rakic, 1990; Geschwind and Rakic, 2013; Kolk and Rakic, 2022). There is subsequent tangential (lateral) propagation of thalamic inputs within the cortical network, but the majority of intracortical axonal connections just leads reciprocally to adjacent cortical regions, either within the gray matter or just below it as arcuate fibers.

The associative cortex is usually divided into regions classified as unimodal when interfacing with a primary sensory/motor region, and as bimodal and higher order polymodal association cortex (Figure 4). This implies a tangential processing order from sensory inputs to higher cognitive functions (Sydnor et al., 2021). However, other views are possible as discussed by Lipp and Wolfer (2022) (Supplementary Figure 1). If one considers the limbic cortex as the evolutionarily oldest associative cortex, the processing order gets reversed: signals from hypothalamic and basal forebrain nuclei are processed in the limbic cortex and provide from there continually tangential information to the associative cortex. These signals include the needs for survival and reproduction: food and water intake, health and stress status, motivations and emotions, social and territorial behavior, reaction to threats, and to chemical information. The tangential impact of the limbic system on higher order associative cortex is doubled by the unidirectional mammillo-thalamic tract, from a region known to elicit fear and aggressive responses when stimulated by electrodes (Lipp and Hunsperger, 1978; Canteras, 2002). In humans, the size of the mammillo-thalamic tract is comparable to that of the fornix, estimated to contain also one million axons (Tomasch, 1969) or to the optic tract (Jonas et al., 1992). Fiber bundles of this size usually indicate important functional properties. But the same site sends a bifurcating projection, the mammillo-tegmental tract, to the motor and central gray parts of the midbrain and pontine areas, paralleled by the septo-habenular pathway. These regions harbor most nuclei of the ascending monoaminergic systems (noradrenergic, dopaminergic, serotoninergic) and can also activate the intralaminar thalamic system able to highlight wider cortical areas. Thus, a wide network of subcortical structures provides both the anterior ventrolateral/dorsomedial thalamus but also the midbrain and pontine tegmentum with status copies of subcortical activities. This arrangement (plus the lateral input from the limbic system) ensures that the higher-order associative and specifically the prefrontal regions remain under the ascending control of the basal motivational systems (Averbeck and Murray, 2020) while using simultaneously all types of associative cortex for preparing appropriate motor output of their hemisphere.

### The role of long intracortical connections of the associative cortex

There are two types of long intracortical connections orchestrating spatial patterning of neuronal activity as reflected by the various imaging methods. One is for rapid propagation of signals connecting reciprocally distant regions, in primates linking posterior visual zones with frontal motor regions along two fiber streams. They probably developed for eye-hand coordination in an arboreal habitat but were modified in humans for increasingly co-transmitting auditory input. The other long connections of the associative cortex include the anterior commissure and the corpus callosum (CC) interconnecting the two hemispheres, partially symmetrical (homotopic) and partially asymmetrical (heterotopic), as can be seen in De Benedictis et al. (2016). Somewhat surprisingly, the presence of long intracortical axons running in the white matter is sparse as estimated by tractographic methods (Rosen and Halgren, 2022) even when considering the roughly 200 million axons of the CC (Aboitiz et al., 1992). In other words, neighboring cortical areas are much stronger interconnected than distant ones, as connective strength decays exponentially with distance (Rosen and Halgren, 2021), indicating that the primary mode of cortical interactions and propagation occurs between or along neighboring regions.

### Cerebellar feedback loops

For both language and movements, the most important subcortical input to the associative cortex is provided by the cortico-cerebellar feedback system. About 20 million fibers per side leave the cortex along the internal capsule abutting into the corticopontine tract (Tomasch, 1969; Figure 2). This number corresponds roughly to some 10 percent of all long descending white matter connections in the human cortex. But most of these axons end in the pontine gray nuclei, which in turn send ascending and widely divergent projections to the contralateral cerebellar hemispheres. Their output converges then again to the cerebellar dentate nuclei and reaches the ventrolateral thalamus somatotopically through the side-crossing superior cerebellar peduncle (Supplementary Figure 2). Thus, cortical activity is a dynamically fluctuating pattern shaped by the parallelly arranged entity of cortico-ponto-cerebello-dentato-thalamico-cortical loops. If cognition is linked to activity of associative cortices as generally believed, the cerebellum automatically becomes a fundamental part of cognitive processing. In simple terms, all associative cortex regions receive a variable blend of somatotopic (“homunculus-shaped”) cerebellar status copies of ongoing motor output and non-somatotopic limbic motivational signals from two sources. Such dynamic blending biases the probabilities of the next patterning action taking place in the main cortical homuncular zones.

## Dividing the associative cortex into audiomotor and visuomotor networks (3)

Given the apparently equal size of the disproportionate output systems in the motor cortex, each one with a bundled output system, one must assume that most of the associative cortex area is functionally divided accordingly. Details will be described later under sections “Functional properties of the language cortex” and “Thinking properties of the visuomotor cortex,” but it seems obvious that the language network must handle an extremely high working load (Fallon and Pylkkänen, 2024; Regev et al., 2024) that enforces connective specialization. Here we will deal first with historical attempts to map the associate cortex according to language, culminating in numerous neocortical network models.

### The topography of the language network

**FIGURE 5:**
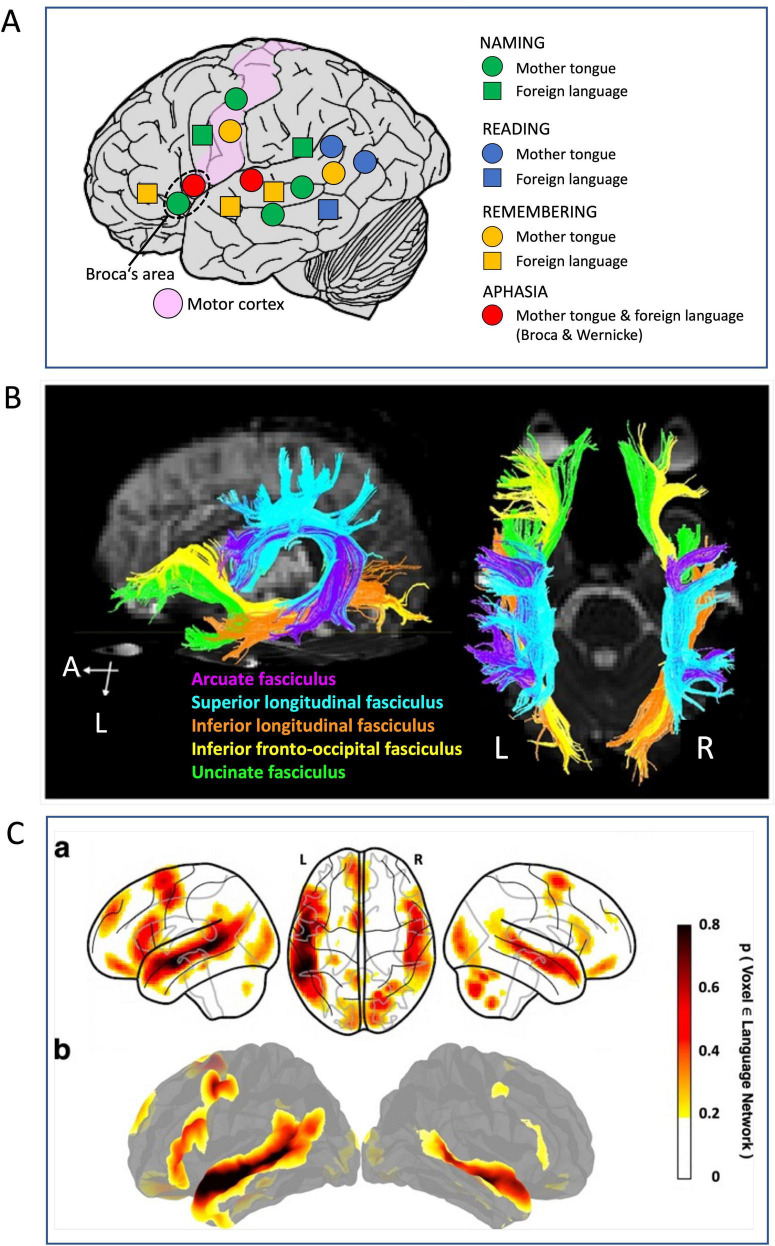
Old and new methods for visualizing neocortical language networks. **(A)** Language mapping by pre-operative electrical stimulation inhibiting language zone according to Ojemann (1983). The only loci inhibiting all aspects of using mother tongue or foreign language were Broca’s and Wernicke’s areas, while an extended map of inhibitions showed involvement of visual and temporo-parietal and premotor, prefrontal associative cortex but also motor cortex. Drawing showing principle only, no real data. **(B)** Tractography by means of diffusion tensor imaging showing the main fiber tracts underpinning the cortical language network. There are two bundles forming a superior stream, and three a ventral stream. Details in text and in the original paper (Jang, 2013). The figure has been slightly modified, under Creative Commons Attribution License 4.0. **(C)** Parts of a probabilistic functional atlas using fMRI data from 806 individuals (Lipkin et al., 2022) revealing those portions of the language network supporting high-level language comprehension and expression. Details in text and original publication. Modified under Creative Commons Attribution License 4.0.

This is probably the anatomically and functionally best analyzed network (Figure 5). Its kernel was revealed first by Bouillaud who reported in 1825 that lesions in the frontal lobe impaired the ability to generate words (Stookey, 1963). Broca (1861) followed up much later but eventually established the left inferior frontal cortex as important structure and succeeded in acceptance of the concept by the scientific community. Sensory aphasia was then reported by Wernicke (Lanczik and Keil, 1991) after damage to the temporal auditory associative cortex and the observation of the arcuate fasciculus linking reciprocally the two regions led soon to the insight that language was based on an intracortical network that probably included other associative cortex regions as well (Geschwind, 1970).

Functional mapping started with placing stimulating electrodes on the cortical surface and occasionally in the thalamus of awake patients answering questions and naming objects (Figure 5A). The stimulation elicited inhibition of ongoing cortical processing that became apparent as temporal inability to speaking, listening, memorizing, and naming words (Ojemann, 1979, 1983). This implied that widespread other cortical regions were involved in language processing (Friederici and Gierhan, 2013). A tractographic summary illustrating this (Figure 5B) has been provided by Jang (2013) showing a dorsal stream responsible for phonation, namely the arcuate fascicle and the superior longitudinal fascicle whose various components are interconnecting frontal, occipital, parietal and temporal lobes (Dick and Tremblay, 2012). It is believed to support sensorimotor integration such as repetition of words or sounds in speech. The second more ventrally situated fiber system is thought to subserve auditory comprehension and recall and planning of talking (Castellucci et al., 2022). It is embedded in the myelinated fiber sheet of the extreme capsule and includes three fascicles: inferior longitudinal, inferior fronto-occipital, and uncinate. The latter connects inferior prefrontal cortex with limbic cortex transferring impulses to and from the amygdala and the ventral hippocampus. In a dorso-basal view, hemispheric asymmetries in these fiber bundles are apparent, the dorsal complex appearing denser at left, and the inferior tracts more developed at right.

An overall view of functional activation of the language network is reported in Figure 5C showing parts of a probabilistic functional atlas using fMRI data from 806 individuals (Lipkin et al., 2022). Like the tip of an iceberg, it shows just the top 10 percent of fMRI records visualizing peak activations during identifying the fronto-temporal language network that selectively supports high-level language comprehension and expression, including word meanings and syntactic/semantic organization. However, the laboratory-specific design of the language-related tests excluded simple perceptual processes and motor elaboration, thus likely concealing the real extension of the language cortex which might resemble more the mask of the language cortex as shown by Fallon and Pylkkänen (2024). Its asymmetry patterns reveal some interesting points. They coincide roughly with the tractography in Figure 5B but show asymmetrical activity of Broca’s area, large at left and small at right. Similarly, the posterior activation zones in the right hemisphere appear to coincide with the tractography data. In any case, a detailed tractographic analysis of cortico-ponto-cerebellar circuitry as related to language has just begun (Yin et al., 2023) while the feedback of the cerebellum to the associative cortex as indicated by Palesi et al. (2015) deserve specific future attention as well. Clearly, the language network further controls prosody and emotionality of speech and song by including the basal ganglia and cerebellum (Wildgruber et al., 2006; Ceravolo et al., 2021). Likewise, participation of hippocampal circuitry in generation of words is likely (Knecht, 2004; Duff and Brown-Schmidt, 2012; Alamri, 2017; van de Ven et al., 2020; Gattas et al., 2023).

### Functional properties of the language cortex

#### Prosody and melody: language as a musical instrument

The mastery of voice is one of the top achievements in human evolution and has high output demands for the corticobulbar tracts. Anatomically, this must be achieved by extremely precise opening and closing of the vocal folds by the crico-arytenoid and intrinsic arytenoid muscles, and by the cricothyroid muscles adjusting pitch (Belyk et al., 2018; Lu et al., 2023), while the internal muscle fibers in the fold, the embedded musculus vocalis, regulate tension and degree of vibration. In comparison to the skeletal system, these are relatively few muscles for subtly moving few cartilages. Nonetheless the motor cortex holds a double representation of the laryngeal muscles (Simonyan and Horwitz, 2011; Simonyan, 2014; Belyk et al., 2021; Taheri, 2024), although it has been questioned whether the lowest representation is not located in premotor cortex (Eichert et al., 2020). The second challenge to deal with is the modulation of the produced sound by a complex system of about 100 muscles (Ackermann and Riecker, 2004), including the pharyngeal muscles, the hyoid cartilage, the jaw, the tongue, and the facial muscles that together form a rather loose and flexible system without the typical bone-to-bone configurations as in the locomotor apparatus.

Moreover, the entire sound producing and controlling machinery is innervated by different cranial nerves whose impulses must be perfectly synchronized. To complicate matters further, the corticobulbar tract decussates only to 50%, making it difficult to disentangle the role of left and right tracts, and the sensory feedback through thalamus and cerebellum must operate equally fast and arrive precisely timed at the primary sensorimotor cortex. This is no trivial task, putting further functional load on the language part of the cortical homunculus. Hence, it makes functional sense to have the language system strongly lateralized, leaving to the opposite hemisphere only special functions such as providing prosody, melodiousness, and thereby emotional content (George et al., 1996; Wildgruber et al., 2005; Ross and Monnot, 2008; Patel et al., 2018; Ceravolo et al., 2021; Mauchand and Zhang, 2023).

### Perfect singing

**FIGURE 6:**
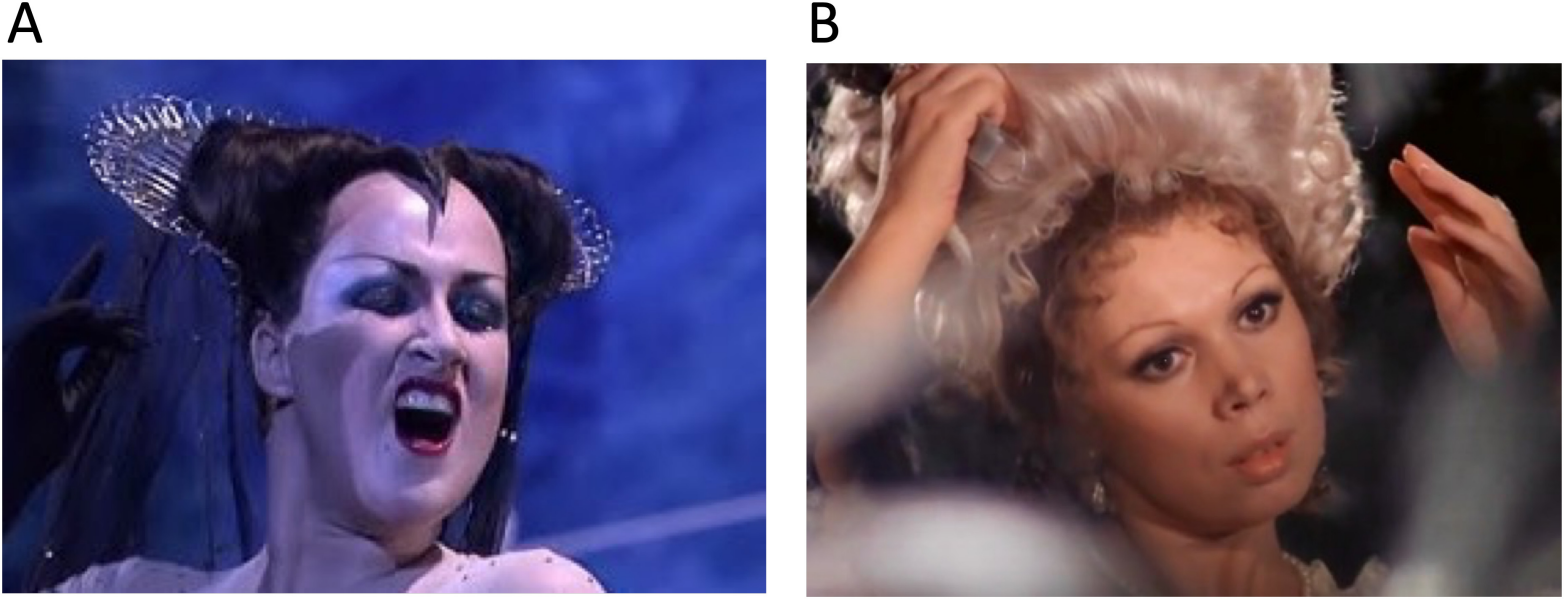
Extremes of human song performance. **(A)** Diana Damrau in Queen of Night, Opera “The magic flute”, by W. A. Mozart. Note absolute mastery of vocal muscle movements in face, mouth, tongue, pharynx, larynx and thorax but no melodiousness at the highest pitches. Vocalization is highly aggressive and frightening the listeners”. To see and hear the video (with permission from Unitel), click this link https://youtube.com/clip/UgkxS0tjDir8ZsRKGMcoCxzMXyN5RY1dhB5z?si=e2Bc6bYkx_DNAbyy. **(B)** Slow legatos, much prosody and smiling face in a love song by Mirella Freni, Opera “Figaro’s marriage” by W. A. Mozart”. To see and hear the video, click this link https://youtube.com/clip/UgkxwnN99AMXx2bzKSINto-hTPYtqAdTdEmc?si=_YoI6FhDQQ_Zzk01. To download both videos for permanent storage, see the Supplementary material.

This is illustrated by comparing arias from two Mozart operas that show extremes in controlling the vocal apparatus, using videos from the “Magic Flute” and “Figaro’s Marriage” (Figure 6). The famous aria of the queen of the night with extreme staccato pitches requires perfectly coordinated movements of trunk, chest, larynx, pharynx, tongue, and facial muscles. Yet there is hardly any melodiousness at all, and the emotional effect for the listeners is usually hair-rising fear as intended by the composer. Other Mozart arias sung by lyric sopranos show a very different mastery of the vocal apparatus when performing a love song (“Deh me vieni non-tardar”, “Come, do not delay, oh bliss”). Yet here the voice has many overtones, a slow melodious legato and much specific prosody, sending a message of social attraction even when the language components are not understood or distorted (Albouy et al., 2020). Another aspect from these arias is the perfect synchrony of the facial muscles underlying emotional mimics such as smiling or aggression, even though singers can train their vocal apparatus without emotional involvement.

Neuroanatomically, it appears that the difference between the two arias reflects the degree of callosal interaction when the language-dominant hemisphere suppresses the activity of the contralateral hemisphere during preparation and execution of the song. The queen’s aria is so demanding for the control of the vocal tract that any movement impact of the opposite hemisphere must be blocked, specifically in the most difficult parts at highest pitch. On the other hand, Susanna’s rose aria sounds lovely-emotional when the dominant motor system relaxes contralateral inhibition and allows the opposite hemisphere to precisely modulate the tonus of the many muscles involved. At the cortical level, there is a small number of inhibitory fibers running in the CC (Rock et al., 2017) offering a morphological substrate for mutual inhibitory control, pending verification in humans.

Taken together, the language system attains a new degree of evolutionary complexity with its ability to maintain two complementary neuronal networks, one preparing and outputting rapidly repeated movements of the vocal tract that require ultrafast sensorimotor and proprioceptive feedback, and a second one, probably in the non-dominant hemisphere (Albouy et al., 2020; Ross, 2023), generating rhythmicity, prosody and melodiousness by subtle control of non-moving muscles. One may note that the intensity and variations in prosody can additionally change the meaning of the semantic content even without melody: “deh me vieni non-tardar” sounds attractive in soft voice (“please come my dear”), becomes signaling a neutral request in moderate tone (“it’s time to come, my dear”) and transforms to an aggressive order when spoken in loud volume (“now come my dear”).

Thus, the language output systems also provide the framework for sending and receiving highly differentiated emotional signals, creating a simple form of telepathy accompanying the word machinery yet powerful enough to synchronize or manipulate feelings and activities of partners or groups (Nowak et al., 2017; Thomas and Kirby, 2018; Hughes and Puts, 2021). But is this related to a specific way of thinking?

## Two ways of thinking? How the two cortical motor output systems may shape different thinking (4)

There seems to be no generally accepted definition of thinking. According to the motocentric view of this paper, thinking can be derived from the activity patterns of ongoing neuronal actions in the motor cortex. Within the networks of the associative cortex and its subcortical connections, this generates virtual action (movements and/or visualizing scenarios requiring it) not translated into movements, comparable to the suppression of actual movements during dreaming (Senzai and Scanziani, 2022). Virtual memories can be generated from kernels or fragments of old memories, a process known as confabulation (Gilboa, 2010; Bateman et al., 2024) and combined with virtual actions. Depending on which networks are dominating, there will be visuomotor or audiomotor thinking modes that require flexible switching and interactions, provided that both networks have comparable impacts (Alderson-Day and Fernyhough, 2015; Amit et al., 2017; Komarla, 2024). While thinking modes must be inferred, learning styles of children provide a hint at underlying brain networks. Silverman (2002) found in 750 children about 33% primarily visuospatial learners, about 25% strongly auditory sequential learners, and the other learners mixing both modes according to the degree of assumed hemispheric lateralization, but large-scale scientific studies in school children appear missing.

### Functional limitations of language-related thinking

Language-bound communication and thinking has one strong feature transcending personal interactions, namely the power to reduce and encapsulate information, specifically linking words with simple visual information (Pulvermüller, 2023). On the other hand, while perfect for transmitting both social and contextual information, likely colored with emotionality by the other hemisphere, the language system with its functionally demanding double output appears linear in time and heavily dependent on precisely timed cerebellar feedback (Fallon and Pylkkänen, 2024). A typical speech or talk usually lacks the dimension of time and appears linked to the present, intended to instruct, or convince listeners to follow some ideas and opinions, generally with some level of emotionality but never gives the impression that it could diverge into unexpected intellectual directions. Short lasting exceptions are provided by satirical comedians playing with expectations of the auditory, while long-lasting intellectual effects can be generated by speakers using sarcasm yet only in listeners with equally high intellectual capacities (Huang et al., 2015). During small talks or in informative conversation, the emotional content of the exchange of word strings appears moderate and the intellectual load minimal (Dunbar, 2012; Tomasello et al., 2019; Castellucci et al., 2022). Language itself appears inefficient for sketching complex relations in scenarios unfamiliar to the listener and needs support by accompanying simple facial movements signaling more complex emotions. Other support to a narrative is provided by telling familiar examples, in written form also by illustrations of varying complexity.

There is strong evidence that thinking without talking can be observed in fully aphasic patients (Fedorenko et al., 2024a) while simple observation of human behavior suggests that talking with minimal thinking is a common phenomenon. Verbal thinking seems to occur in many persons, resembling serial monologuing, sometimes even muttered (Alderson-Day and Fernyhough, 2015; Amit et al., 2017; Grandchamp et al., 2019). Such loud thinking is helpful in dissecting various aspects of a problem but appears ill-suited for rapid executive planning of different actions.

#### Human ethology: words as bullets or weapons

While listening to a speech of a politician in a foreign language, the spoken output structures (phonemes and words) resemble the rapid charging of an automated gun with various types of simple verbal ammunition. Since the fast cerebellar feedback is projected to different parts of the associative cortex, the selection of the next output is nearly automatic: one word gives the next one, following the language attractor in the motor cortex. Overall, the main cognitive load is small as the phonemes to select from are rather similar but may contain different emotional values priming a choice of words. Visual information is co-processed but preferably in form of simple symbols such as cartoons or emojis (Garagnani et al., 2021; Dalle Nogare and Proverbio, 2023). Hence the main cognitive load of talking is to provide understandability and emotional content, and to adjust this to the expected level of comprehension in the recipients. This is demanding but does not require superior executive abilities. With respect to verbal thinking, the permanent cerebellar feedback may lead to another phenomenon: the first sentenced output reflects a belief - you say what you believe, but the feedback initiates a vicious circle – you believe what you say.

Another ethological aspect to consider is language used in debates. Here, language resembles a tool to dominate the views of other persons, itself a simple demonstration of personal power. Given the linear output structure of the verbal string supposedly reflecting an argumentative thought, it is difficult to see how different mental scenarios become integrated in a string of words during a debate. Quite likely, the narrative conveys a simple contrast strategy based on opposing properties such as black or white, in other words, the essentials of dialectical thinking. In any case, the impact of hypothalamic inputs on this form of using language is obvious and may drive the person-directed approach in rhetorical debates designed to produce a winner or loser.

The least cognitive verbal output is cursing, a string of depletive words and sentences without target, or insultingly crying and shouting at persons as a substitute for physical aggression (Pisanski et al., 2016; Stapleton et al., 2022). Part of this unfiltered verbal output may reach the vocal system from the cingular cortex via the periaqueductal gray (Jürgens, 2009) or it may result from poorly inhibited thalamocortical throughput as in more complex tics of the Tourette syndrome known as coprolalia (Gates et al., 2004). Quite common are English four-letter words while other languages are verbally more inventive.

A last property of the verbal thinking network is its extreme sensitivity to simple trigger words evoking positive or negative emotions that likely prime the course of further thought (Feng et al., 2021). Politically, the most dangerous version is known as absolute words symbolizing unchangeable meanings linked with aggressive risk-taking (Mehravipour, 2025). The obvious link with facial mimics underscores the intrinsic integration of outputs of the linguo-oro-facial homunculus but indicates also the presence of a network linking simple visual objects with audiomotor output.

#### Combining language and movements

The use of language does not preclude movements of arms, hands, and legs. In fact, sign language can successfully replace talking when normal verbal output is impeded, which has led to the early hypothesis that language is a derivative of the manual non-verbal system (Vicario, 2013). But during ordinary talking they appear rarely oriented, even in gesture-using cultures, or partly suppressed when language or song is intensely expressed. In political speeches, movements of hands and arms resemble symbolic fighting (hacking, sweeping, pointing to imaginary adversaries) but lack finer timing or complex structure. This is what one would expect from partly competing neural networks, but special cases are obvious. Musical conductors silently control the output of a symphony orchestra by arm, hands, and expressive body movements. Likewise, word-gesture combinations for pragmatic intents are seemingly improving brain correlates of verbal comprehension (Tomasello et al., 2019). Another combination of language and movements is texting on smart phones, “Fingered speech” (McWhorter, 2013), phylogenetically considered as a new form of linguistics replacing spoken output whose underlying neural networks have not been analyzed thus far.

### Thinking properties of the visuomotor cortex

Essentially, much of the visuomotor cortical network is defined by the connection pattern imposed by the corticospinal output system controlling the upper extremity, specifically hand and thumbs as symbolized by the cortical homunculus in Figure 1. In terms of complexity, this system is (comparatively) simpler than the language network and its output system, as it is based on a phylogenetically old locomotor system developed for displacement of bones through attached muscles, while the foundations of its proprioceptive control through ascending tracts and cerebellum have evolved in parallel, being coordinated rather perfectly by the vertebrate midbrain for many hundred million years. Thus, what could have been the reasons for developing a second associative cortical network centered on the use of arms and hands under guidance of the motor cortex?

#### Multidimensionality of movements

A first reason is proximal and distal dimensionality. Arms and hands can move in three dimensions for manipulating objects, giving raise to body-based measurements (Kaaronen et al., 2023). Locomotion itself, combined with visual, auditory, and proprioceptive feedback provides measures of space and time. For example, picking up a distant moving object requires estimation of trajectories of the own body and that of the moving object, plus a sense of time derived from locomotor experience such as the ability of extrapolation in animals and humans studied by the Russian school of Krushinsky (Poletaeva et al., 1993; Poletaeva and Zorina, 2015). Since modern functional brain mapping limits active movement, it might be difficult to visualize how an audiomotor cortical network transforms these basic and ongoing operations into real-time executive properties involving planning and navigation. But there are at least examples of functional brain mapping indicating negatively correlated linguistic and non-linguistic networks (Quillen et al., 2021), and multiple-demand (MD) networks only partially overlapping with language network have also been demonstrated (Duncan and Owen, 2000; Fox et al., 2005; Fedorenko et al., 2013).

Non-linguistic networks appear to be also involved in orchestrating multiple parallel or complex outputs difficult to handle by the language system. For example, a chess game can be connotated by the sequential movement of figures (e.g., pawn e2-e4) sufficient to replay the game. However, chess champions can play against many opponents (up to 50) without seeing the actual chess boards. Yet it appears unlikely that they just recollect the serial notation of the many moves having been played by the opponents, as one would expect if he were using his language network primarily. On the other hand, chess grandmasters can visually recognize 50,000 – 100,000 of positional templates yet handling them in chunks, that is groups of pieces in tactically meaningful clusters (Gobet and Simon, 1996; Gobet, 1997). This ability is co-mediated by the fusiform gyrus (Bilalić, 2016), indicating that chess playing is ultimately a visuo-motor process, the final output being a simple motor act, either moving a piece or making a mouse click.

Imaginary movements, mental grasping and shuffling of complex objects and their relations seem more related to the arm/hand homunculus and its network derivatives than to the language networks. Both networks certainly share components, but it seems difficult to conceive the language network in a commanding role for complex executive-cognitive operations. For example, communicating complex scenarios and mathematical problems by words alone seems hardly possible even in written form. Likewise, manual creativity such as painting or sculpting a wood figure, or splitting a flint stone, requires transformations of visual imagery into precise manual actions. Another activity-dimension of the manipulating network could involve translating narratives into visual images in form of a movie. But the common denominator of these examples is that they cannot be done with words.

Creating art by words alone is clearly possible provided it has an understandable narrative that plays with words, intonations and rhythmicity evoking memories and emotions in listeners, specifically in those that have a similarly well-developed language network. But it needs someone reading or hearing it, while creating a piece of art or a new technique does not necessarily need social partners.

#### Weaknesses of the visuomotor system

On the other hand, the visuomotor network has weaknesses itself. For one, it depends much on visuospatial support to define virtual goals and approaches. An fMRI study has shown that mental imagery of goals and movements indeed activates motor networks (Szameitat et al., 2007), implying that during thinking or daydreaming the brain remains in a stage of visuomotor activity without generating movements (Golchert et al., 2017). This most likely requires some of the mechanisms used to suppress active movements during REM sleep and dreams, as potential motor output is often generated but blocked at the brainstem level or thalamus by inhibitory GABAergic and glycinergic networks (Brooks and Peever, 2012), thus remaining virtual. Hence, two problems emerge. Firstly, transmission into executive actions remains suppressed since higher motivation and physical efforts are needed, much more than by talking. Even though active daydreaming is thought to correlate positively with creativity, poor attentional control results in drifted thoughts and poor creativity (Sun et al., 2022). Mind-wandering can even be associated with adverse effects such as anxiety disorders (Fell et al., 2023), resembling the nightmare situation when intended motor escape actions from a threat do not generate any proprioceptive cerebellar feedback, thus instilling growing panic and sudden awakening.

The preceding sections laid out the design, functionality and partial incompatibility between visuomotor and audiomotor networks. The following sections try to explain the various evolutionary processes leading to the hypothesized enlargement of the language network. It is thus necessary (i) to explain in some detail the presence and origin of genetic variability in cognitive networks of the mammalian and human brain, (ii) to provide a plausible scenario for the evolutionary changes in anatomy and networks, and (iii) to examine how these changes led to cultural transformations acting as new selective agents.

## The forebrain as a playground of evolution: individual and genetic variation of networks (5)

It is conceivable that a large forebrain, specifically the associative cortex, does not only provide intelligence but buffers mutations influencing its connectome, for the simple reason that they can be often compensated by developmental and adult plasticity protecting the carriers from natural selection (Lipp, 1988, 1989, 1995; Lipp and Wolfer, 1995, 2002). In lenient environments forebrain-related mutations accumulate and provide a pool for natural selection, at least as far the building blocks of the brain such as neurons, glia, neurotransmitters, and others more remain intact. Likewise, natural selection eliminates rapidly mutations that cause damage to primary sensory or motor systems. Thus, evolutionary adaption of forebrain and associated behavior but without changes in body morphology can occur after massive environmental changes in much shorter time spans than generally expected, as in the hippocampus of feralized mice, where this evolutionary adaptation can take place after just a few generations (Lipp and Wolfer, 2013). Important to note here is that gene variants never encode behavior of any type but just alter the probability of its appearance as patterned movements. But is there evidence for individual and human genetic variation of brain traits and corresponding functional changes, specifically with respect to language?

### Size variations of specialized neocortical areas and networks

Behavioral evolution in animals works primarily by selecting individuals showing observable situation-specific motor characteristics. Thus, behavioral traits are the tokens of evolution whose selection entails appropriate sensory or physiological adaptations. But behavioral traits should be predictably observable over time to permit natural or sexual selection. For example, individual behavioral traits such as slow or rapid moving may bring evolutionary advantages in open or forested habitats, but if each member of a population shows unpredictably the entire spectrum of locomotor speed, there cannot be selection for extremes. As humans have two motor output systems with limited capacity of operating simultaneously, visuomotor and audiomotor outputs usually alternate effortlessly if the underlying networks are functionally balanced. Thus, such unpredictable switching impedes classification by observation and therefore prevents or slows natural selection processes.

On the other hand, simple observation and folk psychology indicate that there is a spectrum of persons more inclined to the use of language, the “talkers” than others being less verbose yet being more practically oriented, the “doers”. The most extreme cases of talkers include neuropathological logorrhea (Pomales et al., 2023), a never-stopping avalanche of words that may also follow an episode of emotional overload. Complete lack of talking with an intact motor cortex, mutism, may indicate damage to Wernicke’s area (Aggarwal et al., 2010). However, not being talkative can have many causes. Just by observing ordinary persons during their daily activity, one might expect that managers, commanders, most artists, athletes, scientists and manual workers minimize talking when trying to do something, while constant talking seems to interfere with most practical and goal-directed activities. This implies substantial structural and functional variation across human brains underlying such personality differences.

Indeed, the neocortex shows substantial individual variations in the extent of cytological subfields, the best investigated of which is the size of the primary visual cortex that shows a two- to threefold variation, correlated with size of optic tract and lateral geniculate body (Andrews et al., 1997). Functionally, these size variations correlate positively with superior visual abilities (Song et al., 2013; Verghese et al., 2014). Likewise, the planum temporale, a part of the temporal language cortex, often shows sizable asymmetries visible during brain dissection even though the functional meaning of these asymmetries in language and psychiatry is open to various interpretations (Galaburda et al., 1987; Rossi et al., 1994; Amunts et al., 1999).

**FIGURE 7:**
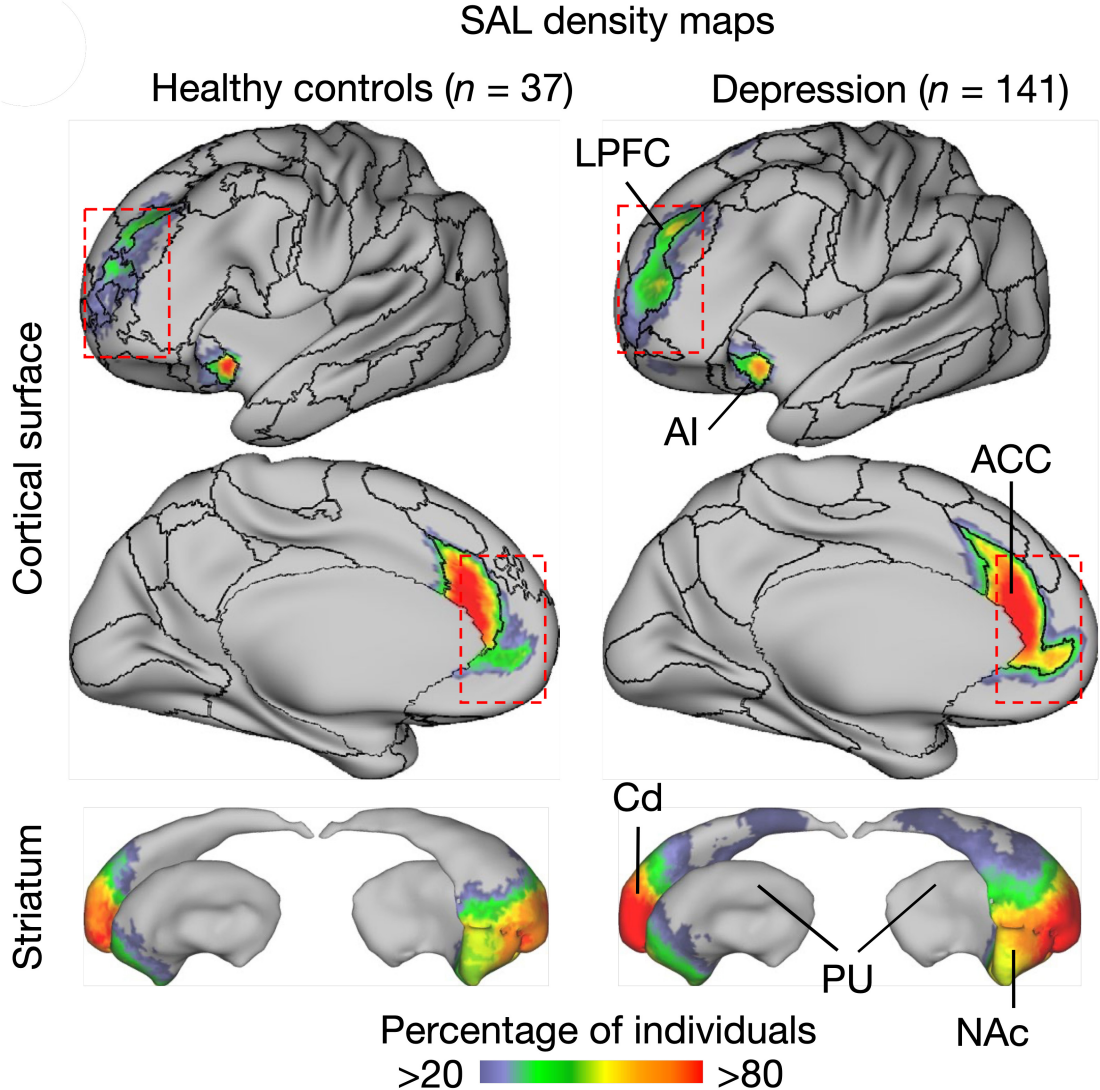
Visualized default mode salience network shows stable long-term size variations in depressive and healthypeople (Lynch et al., 2024). The significance of these findings is that they reveal a network as a trait, that is a constant individual characteristic of a given person. The graph shows a figure from the cited publication where further details can be found. SAL, salience network, ACC, anterior cingulate; AI, anterior insular cortex; Cd, caudate nucleus; HC, healthy controls; LPFC, lateral prefrontal cortex; NAc, nucleus accumbens; PU, putamen. Copied under Creative Commons Attribution License 4.0.

In terms of functional brain mapping, related phenomena have been observed. Ren et al. (2021) compared the auditory (AC) and visual cortex (VC) areas in monkeys and humans and found that interindividual variability was larger for the AC than for the VC, and that such variability was largest in associative AC areas. Likewise, depressive persons were undergoing multiple fMRI mapping for a salience network (default mode network) thought to reflect homeostatic body needs in non-thinking mode (Seeley, 2019). This study showed a systematically larger region of this network in depressive as compared to non-depressive people (Lynch et al., 2024) and Figure 7. Both studies strongly suggest that size variation of cortical networks can be a trait, a constant behavioral or neural characteristic of an individual. Indeed, recent studies have shown heritability of individualized cortical network topography to be greatest in prefrontal, precuneus, and posterior parietal cortex (Anderson et al., 2021).

It appears therefore plausible that size of cerebral hubs or connective variations of language and non-verbal networks underlie corresponding behavioral phenotypes, and, accordingly, their way of thinking as indicated by magnetoencephalography (Nishimura et al., 2015). Thus far, there seems to be no study showing whether the language hub or the manual hub show relative differences in size within the motor cortex. To follow the hypothesis of this paper however, there should be genetically differences in extent or connectivity of the two networks that could be subject to natural selection. How this might happen can be illustrated with two examples from developmental neuroanatomy.

### A tipping point in prenatal brain development profoundly reshapes the cortical connectome

As documented primarily in in mice and humans, there are individuals lacking a CC and with that an important fiber connection between the left and right hemispheres, mostly between associative cortex (Figures 3, 4), but the fibers also include axons from the contralateral claustrum (Milardi et al., 2013) and other fibers of passage. Besides a completely missing CC (callosal agenesis), the older literature as summarized by Lipp and Wahlsten (1992) reports many neuropathological cases with diminished CC (callosal dysgenesis), usually associated with other malformations such as cerebellar dysplasia, and the CC of normal persons shows strong variations in size. But how can this large tract in the human connectome show extreme variations?

**FIGURE 8:**
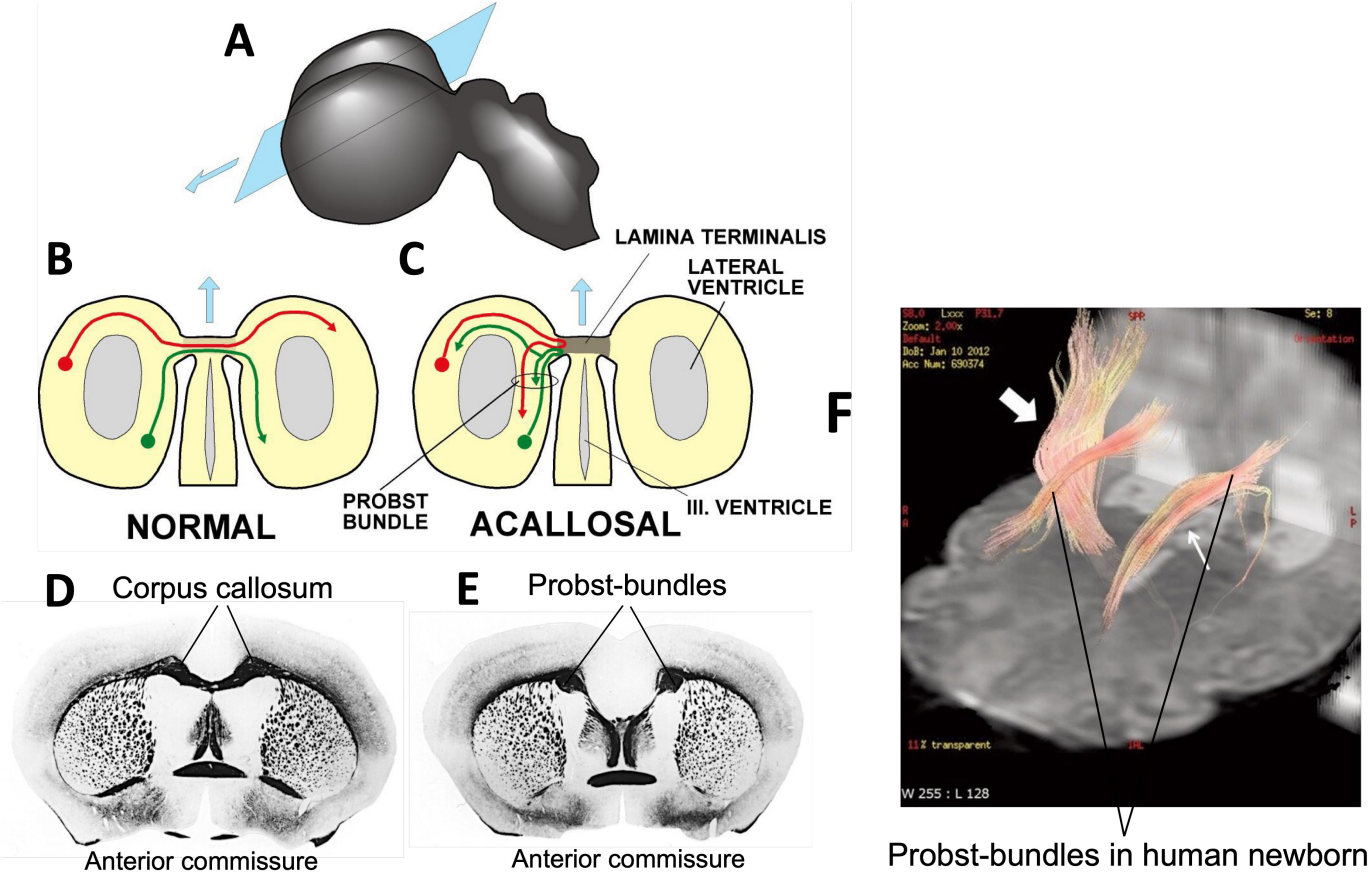
Massive developmental rerouting of callosal connections in mice and humans with missing corpus callosum. **(A)** Section plane through the embryonic forebrain vesicle. **(B)** Normal embryo in which fibers cross a thin bridge of glial cells called lamina terminalis. **(C)** Premature closing of the glial bridge (caused by many factors) blocks callosal fibers and forces them to seek neurons to which they can connect to avoid cell death. **(D)** Coronal section from a mouse brain with normal corpus callosum stained for myelin (Gallyas stain). **(E)** Same plane in an acallosal mouse with distinct aberrant Probst bundles. **(F)** Tractography of an acallosal human newborn (Pereira et al., 2014) showing longitudinal Probst bundles (small white arrow) and a normal corticospinal tract (large white arrow). These images show the ease by which the mammalian connectome can be reorganized due to developmental plasticity. Copied and modified under Creative Commons Attribution License 4.0.

The process is illustrated in Figure 8. Showing an early stage of brain development with the vesicle forming the telencephalon (Figure 8A). The rostral end is formed by a glial sling that serves as bridge for fibers crossing to the other side where they usually seek a homotopic position, attracted by regional molecular markers (see also section “Thalamocortical connectivity as a key target in tuning the cortical connectome”). If the sling degenerates too early, the crossing bridge disappears and the axons grow along the midline forming an aberrant bundle named after Probst (1901) to find neurons to make synaptic connections (Pereira et al., 2014; Bénézit et al., 2015). If they fail, this entails apoptotic death of the neuron’s body (Pfisterer and Khodosevich, 2017; Fricker et al., 2018). Early massive overproduction of competing axons and subsequent pruning is one of the main mechanisms in the prenatal development of cortical (and probably many other axonal connections) providing much developmental plasticity (LaMantia and Rakic, 1990). The functional consequences of the aberrant callosal fibers in form of Probst bundles (Figures 8E, F) have remained frustratingly elusive in both mice and human because they reflect a mixture of hemispheric disconnection and unpredictable ipsilateral reorganization (Magara et al., 2000; Siffredi et al., 2013; Lynton et al., 2023).

On the other hand, the tipping point phenomenon provides an interesting case for both neurobehavioral genetics and prenatal diseases. Up to now, about 115 gene mutations have been associated with callosal agenesis (Lynton et al., 2023). From a standard viewpoint of population genetics, callosal agenesis would be considered as a weak trait depending on the added-up contributions of many genes, while the prenatal-developmental view identifies a common critical target, the glial sling, through which subtle genetic alterations but also a maternal illness may entail a multitude of unpredictable phenotypes just because of minor shifts in developmental timing. For an in-depth discussion of this point see Goriounova and Mansvelder (2019).

**FIGURE 9:**
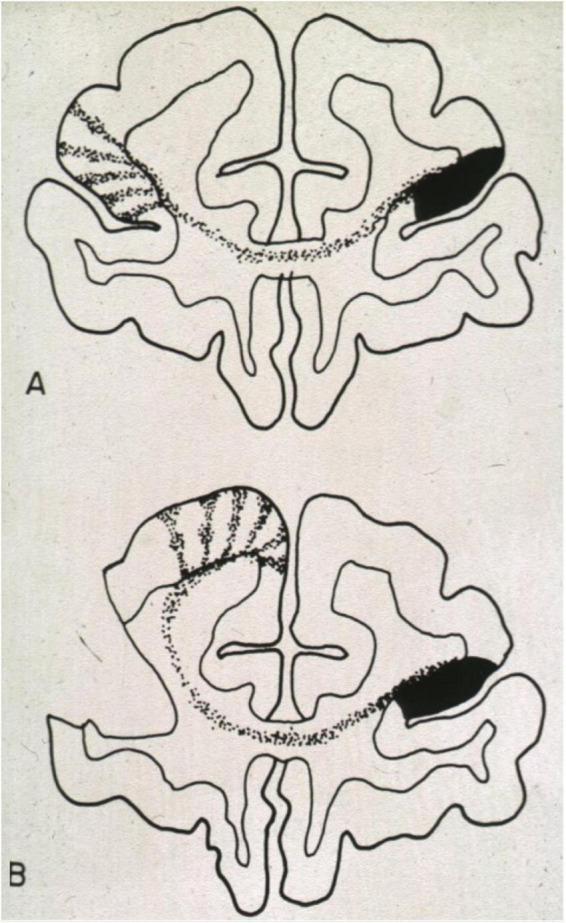
Developmental plasticity of callosal projections after injections of anterograde radioactive tracer into the brain of a prenatal rhesus monkey. **(A)** Injection into a normal brain. Note the columnar dispersal of homotopic and heterotopic fibers. **(B)** Injection of tracer into a prenatal brain in which the usual target region had been destroyed. As in callosal agenesis, the ingrowing fibers seek new contact sites. This will alter the connectivity of those brain regions as callosal and non-callosal (thalamic and cortical) afferents compete for survival of their neuronal body. Figure from Goldman-Rakic (1981), reproduced with permission of MIT Press, Boston Massachusetts.

Figure 9 shows a classic experimental example of prenatal re-routing in Rhesus monkeys (Goldman-Rakic, 1981). After intrauterine injection of radioactively labeled tracer into the prefrontal cortex, callosal axons show primarily homotopic projections to the other hemisphere, arranged in columns interspersed with columns occupied by other afferent axons (Figure 9A). However, after destruction of the genetically assigned target zone, the callosal axons had innervated a clearly heterotopic region (Figure 9B). Of note, developmental plasticity in re-routing axonal connections in the mammalian brain decreases rapidly with the onset of prenatal myelination as the oligodendrocytes produce Nogo-proteins repulsing axonal growth cones (Schwab, 2010). Thus, the re-routed axonal pattern remains spatially fixed, but pubertal or adult plasticity may locally adapt to or mitigate dysfunctions being confined to sites with established synaptic connections. These processes may include dendritic growth or shrinkage (Kolb et al., 2012), synaptic pruning (Huttenlocher and Dabholkar, 1997) or variable myelination of existing connections during puberty (Ziegler et al., 2019).

### Thalamocortical connectivity as a key target in tuning the cortical connectome

Such prenatal developmental plasticity may underlie the development of behavioral and cognitive individuality yet may also produce genetically selectable variants. Early studies by Frost and coworkers focused on the rerouting of thalamic afferents to the hamster cortex after early lesions (Frost and Metin, 1985; Métin and Frost, 1991) and showed that growing retinal axons would connect to somatosensory thalamic nuclei after destruction of their primary targets in the visual thalamus, thereby redirecting visual input to somatosensory cortex, and checked later functionality of these projections (Métin and Frost, 1991; Ptito et al., 2001). A review of further studies on the development of thalamo-prefrontal connections illustrated how the circuitry of the developing prefrontal cortex can be sculpted by a wide range of pre- and postnatal factors (Kolb et al., 2012). Likewise, similar rerouting effects have been suspected to account for visual cortex response in congenitally blind persons (Müller et al., 2019).

From an evolutionary perspective, such shifting of corticothalamic projections seems to be a widespread principle in many species (Molnar and Kwan, 2024). In mammals, the distribution of thalamocortical pathfinding axons appears to be patterned by a genetic distribution of marker proteins such as neural cell adhesion molecules (Enriquez-Barreto et al., 2012), ephrin-A5 and its receptor EphA4 (Dufour et al., 2003), and many more (Bandiera and Molnár, 2022). The variable thalamocortical connections are always accompanied by the development of corticothalamic axons from the same region that self-regulate thalamic throughput through a given nucleus (Figure 3B), providing thereby multiple self-tuning channels not only for sensory inputs to the cortex but many more to the associative cortex. It is thus unsurprising that thalamic throughput regulation has been tentatively linked to schizophrenia (Frost et al., 2004), autism (Ayub et al., 2021), depression (Brown et al., 2017; Lynch et al., 2024) and intelligence (Dear et al., 2024), to name a few.

However, many of these hypotheses are based on deficits in local plasticity within the corticothalamic channels. But if one considers the prenatal re-arrangement of thalamic connections, some other conclusions emerge. For one, developmental competition must lead to a substantial amount of random variation and, accordingly, to differential neurobehavioral phenotypes in the adult population that cannot be explained by genetic or systemic epigenetic factors alone. Genome-wide association (GWA) studies subsume that variation of a specific phenotype is the product of heritable and environmental components. But at least in the corticothalamic circuitry, it appears that it is a triple product of genetic factors, environmental impacts, and random processes. But besides stochastic processes in the formation of connections impacting the associative cortex, there is ample evidence for genetic variations of behavioral traits in mammalian populations. Artificial selection for behavioral and cognitive traits in domesticated dogs (Svartberg, 2006) or rodents (Lipp et al., 1984; Lipp et al., 1989; Perepelkina and Poletaeva, 2022) needs only a few generations to result in divergent phenotypes with altered limbic circuitry (Lipp and Wolfer, 2013). Natural selection works slowly in large populations with variable environment but can speed up markedly after massive environmental changes, specifically if the selection process is accompanied by relaxed genetic control (Gómez-Robles et al., 2015).

## From Pleistocene to Logocene (6)

Therefore, one can assume with some confidence that the period of the last 30,000 years of Homo sapiens was characterized by rapid genetically dependent changes in the connectome of the forebrain. Among other processes, these modulated the vocal system permitting fundamental changes in population structure about 7,000 years ago and which may continue till now. This section shall explain the driving forces behind the evolution of the language network and its impact on changing societies.

### The Pleistocene, a time of maximal dependence on executive brain functions

Quite likely, the demands of living and survival across a 100,000 year period including devastating volcanic eruptions (Hoffecker, 2009; Golovanova et al., 2010) and a peaking ice age (Belknap, 2024) resulted in substantial evolution of brain size and intellectual capacity in both Neanderthals and Cro-Magnon hominins, as the average brain volume of both lines was seemingly larger than in contemporary humans (Lancaster, 2024). Presumably, in these times the dominant motor output system in the cortex was the manual one in either hominine line. The language network certainly existed but might have been somewhat less developed in Neanderthals as implied by a larger cerebellar portion in modern humans possibly related to the presence of more cortico-cerebellar feedback finetuning the language loops (Kochiyama et al., 2018).

Obviously, the visuomotor network was of pivotal importance to get through the various daily activities of hunting and gathering, while the environmental demands left less time for social interactions requiring talk and chatting. Whether in those times singing and prosody in early humans were much developed appears unlikely. Importantly, the language system was tuned to social activities in smaller groups transmitting collected experience but was chiefly of local importance because of the low Eurasian population density during the last ice age. At least in Cro-Magnons, substantial genetic and epigenetic variation of the brain connectome entailing individuals with different abilities including intelligence must have been present. Yet the small group size tolerated quite large phenotypic variation as disabled persons lived for long times and must thus have been supported by family or tribe members in both Cro-Magnons and Neanderthals (Spikins et al., 2018). Likewise, the development of children to sexual maturity appears to be comparable to our time, at least anatomically (Lewis et al., 2024). But what has changed the balance of the neural networks toward the language network? Two factors appear important, namely genetic constellations modifying both the anatomy of the vocal tract and brain development.

### Anatomical factors triggering progress of language

For the appearance of prosody and melody in language, a low position of the larynx enabling resonance of the vocal apparatus was important. The observable descent of the larynx in modern humans yet not in Neanderthals was taken as evidence that Neanderthals could not have developed sophisticated language (Laitman et al., 1979; Laitman and Reidenberg, 1988; Lieberman, 1998; Lieberman et al., 2002; Lieberman, 2007). But this theory is debated since modern children and women have a position of the larynx similar as in Neanderthals without being handicapped in talking, as convincingly pointed out by Clegg (2012). In fact, descensus of the larynx from the neonatal period to adulthood is essential to increase the volume and modulation of the voice, but a descended larynx is also observed in roaring cervids and may simply signal their corresponding body size (Fitch and Reby, 2001). In the same vein, Nishimura et al. (2022) propose that the human larynx became morphologically less complex than in other primates, allowing for improved control of laryngeal cartilages permitting harmonic-rich phonation communicating intent and emotions, while Murphy and Benitez-Burraco (2018) report for Neanderthals the absence of some human genes supporting oscillatory brains processes. Genetically, Gokhman et al. (2020) analyzed methylation maps of skeletal DNA in 63 modern and archaic humans and six chimpanzees and identified a network of 588 face- and voice-associated genes whose hypermethylation state appears unique in modern humans, possibly indicating epigenetic silencing processes.

One may note here that such improvement of the cerebral language network requires not only vocal finetuning but also the integration of the phylogenetically new cortico-laryngeal network with a much older limbic vocal system reaching the larynx via the periaqueductal gray (PAG) and eliciting emotional vocalization such as cries (Jürgens, 2002; Schulz et al., 2005; Jürgens, 2009).

Thus, with the addition of prosody and melody encoding emotions, language became an integral part of prolonged socialization and information transfer in the small societies of the early Holocene, facilitating also the propagation of new technologies (Nowak, 2006; Progovac and Benítez-Burraco, 2019; Hughes and Puts, 2021; Bryant, 2022; Leongómez et al., 2022). However, the same process also laid the ground for its unidirectional and mass-manipulative use.

### Self-domestication and neoteny in late Pleistocene and early Holocene

While both Cro-Magnons and modern humans had long childhood periods, the degree to which their brains maintained juvenile plasticity into adulthood might have been different. There is widespread consensus that the morphological transition of the skull from early Cro-Magnons to modern humans was based on self-domestication although interpretations remain speculative (Benítez-Burraco et al., 2018; Bednarik, 2023). But self-domestication includes many genetic and neurobehavioral changes including neoteny.

Domestication of animals occurs in species having lived in self-chosen proximity with early humans in the late Pleistocene and Holocene, which became tamer either through selective breeding such as dogs, cattle, horses or by living in proximity with humans as cats (Coppinger and Smith, 1983; Driscoll et al., 2009). This process is mostly associated with the appearance of morphological changes in fur color, body size and specific behavioral changes that can be selected by breeders.

Mammalian neoteny is a variant of pedomorphism, defined as the persistence of juvenile traits into reproductive adulthood specifically by slowed development (Encyclopedia Britannica, 2024). Traits can be both morphological or functional, in humans evident as trend to rounded skulls, soft facial features and persisting playfulness. A major common factor underlying these correlated changes in mammals appear to be mutations in the embryonic development of dorsal crest cells influencing adrenal gland function (involved in stress responses), pigmentation, reduction in brain size and prolongation of juvenile behavior (Wilkins et al., 2014; Theofanopoulou et al., 2017; Rubio and Summers, 2022).

Molecular evidence for such neoteny is provided by comparison of mRNA showing that developmental changes in the human brain are indeed delayed relative to other primates by transcriptional neoteny (Somel et al., 2009). Likewise, studies have found evidence for self-domestication by comparing Neanderthal and modern human genomes. Huttner et al. (2024) reported evidence for a human-specific gene set related to three proteins prolonging the metaphase of neural progenitor cells forming the cortex, but these were lacking in the Neanderthal genome yet not in other mammals. In any case, more progenitor cells might have increased the chance of fortuitous new connections. Other mechanisms speeding up variations in human neocortical development might be differences in non-coding DNA regulating the proliferation of progenitor cells such as radial glia (Liu et al., 2025). Thus, genetic variation of genes and non-coding DNA can offer a substantial substrate for accelerating ongoing evolution (Hawks et al., 2007). Self-domestication may have also included discrete natural selection or assortative mating (sexual selection) for behavioral traits (Leach, 2003).

To summarize, archaic human populations appeared to have a fortuitous constellation of (i) developmental programs shaping position of the larynx and hyoid bone, (ii) a network of possibly silenced genes influencing voice and facial features, and (iii) forebrain-specific unselected genetic variation (Lipp, 1995; Deacon, 2009).

Apparently, something must have happened during the Neolithic period and the following Bronze age that triggered a chain of natural co-selection for different gene classes shaping vocal tract anatomy and re-arranging neural networks altering communicative properties of language perhaps into our time. Since human evolution does not forcibly entail higher individual intelligence, an increasing societal impact of the non-verbal properties of language might have contributed to the apparent decline of executive abilities and general intelligence during the last 10,000 years.

### The darker side of the Neolithic revolution

The early Holocene marks the end of the last glaciation period characterized by a climatic optimum (10,000 to 5,000 years ago) associated with a green Sahara (Cheddadi et al., 2021). Increased precipitation promoted the shift from hunter-gatherer societies to early smaller and diverse societies based on various types of lifestyles. This period, known as Neolithic revolution, is traditionally regarded as transition from early agriculture to more sophisticated cultures, culminating in the development of large social organizations such as city-based monarchies. A usual narrative is that humans congregated because of social attraction and willingness to cooperate, reinforced by available food and protection. A less transfiguring and perhaps more realistic view of the Neolithic revolution has developed more recently.

For one, population increase with farming and climatic variations entailed reduced resources and an explosion of violence (Gronenborn, 2006; Fibiger et al., 2023) that culminated in widespread massacres of entire villages, sometimes even associated with signs of systematic cannibalism and selective sparing of young women (Teschler-Nicola et al., 1996; Meyer et al., 2015). Probably this reflected raid-like intergroup aggression led by changing leaders (Glowacki et al., 2016) but not yet systematic and well-organized warfare and aggression. This seems to have occurred with the intrusion of extreme patrilinear warrior societies from an eastern steppe ancestry (Haak et al., 2015) systematically destroying villages and eliminating all men. This process coincided with a massive and geographically widespread reduction of genetic variability of the Y-chromosome in many populations while the variability of female mitochondrial DNA was unaffected (Karmin et al., 2015; Poznik et al., 2016; Zeng et al., 2018). The early appearance of Neolithic fortifications (Parkinson and Duffy, 2007) indicates increasing and coordinated defensive efforts. This would not seem possible without new properties of language and its reception, specifically unidirectional propaganda, and belief in words. However, the Neolithic revolution did not leave written records and there is only one indication that in this period human activity must have been strongly influenced by language capable of synchronizing human behavior at large scales. The presumably first big Neolithic temple of Göbekli Tepe in Anatolia appears to have been built some 9,000 years ago (7000 BC) by people coming together without creating large settlements, was then abandoned after refilling, and partially rebuilt some 1,000 years later (Dietrich et al., 2013). This indicates the existence of mental symbols, beliefs and religion transmitted by language alone but powerful enough to attract – voluntarily or by force - numerous people because the stone pillars were obviously of little practical value and erecting them was costly.

Thus, one of the main Neolithic drivers toward later city-based monarchies was the need for defense and safety. Another was climatic change. The end of the Holocene climate optimum brought global cooling and renewed aridification. In Egypt and other locations, human populations concentrated along available water and agricultural food resources. Evolutionarily almost out of nothing, the city-based high (advanced) kingdom cultures appeared, characterized by organized over-production of food, social stratification and strategic warfare aimed at competing systems (Cioffi-Revilla, 1996; Turchin and Korotayev, 2006; Wrangham, 2018). Instructional language became formalized by written symbols. The appearance of these highly organized social entities was devastating for the other differently developed and smaller social systems: death, slavery or more humanely, strangulating tribute payments. Indeed, these and even modern cultures resemble a transitional stage toward an insect-like superorganism (SO) with strict division of individual functions and highly synchronized activities (Alemi, 2020). This means that humans found themselves integrated in new social systems for which they were evolutionary ill-prepared.

In these new cultures humans had to adapt their lifestyle to very dense populations, spatial and social restrictions and submit themselves to a small elite of rulers. Obviously, this contrasts with the evolutionary profile of humans in the Pleistocene that was based on territoriality, well-developed executive functions for daily life and freedom of moving. Such a drastically altered ecology needed, as always in evolution, not only behavioral flexibility but also neurobehavioral genetic adaptations fitting and stabilizing the new societal structures. Thus, advanced culture became a new genetically selective agent for human evolution (Leach, 2003), facilitated by new possibilities for reproduction such as assortative mating and marriage (Horwitz et al., 2023). The best sign of genetic adaptation to new cultures is the rapid increase of genetic variability in the Y chromosome after the bottleneck in the early Holocene between 7,000 and 5,000 years ago (Karmin et al., 2015; Poznik et al., 2016; Zeng et al., 2018). As the Y-chromosome is linked specifically to male phenotype and behavior, the recovered genetic variability of the Y-chromosome in human population suggests ongoing selection processes on male behavior. Other rapidly advancing genetic adaptations during the last 10,000 years have been observed in response to pathogens, primarily in genes coding immunological protein variants (Fedderke et al., 2017; Kerner et al., 2023). However, these adaptations include also many non-coding DNA variants regulating expression and timing of structural genes (French and Edwards, 2020). Of special interest for behavioral and cognitive adaptations are the human-accelerated regions (HARs), that are highly conserved DNA sequences with human-specific nucleotide substitutions regulating brain development (Liu et al., 2025). It appears therefore rather likely that during the past 5,000 years natural selection has also acted on human behavioral and cognitive traits facilitating integration into the new environments (Molnár et al., 2019; Quinn et al., 2019). But which ones?

## Some neurosociology: dimming the developing brain to stabilize modern societies? (7)

To understand such adaptation, it seems more appropriate to consider some needs of these new social systems by regarding them as human SO composed by primates that are individually driven by hypothalamic rules, namely survival, energy intake and reproduction. In primate social systems, the latter also requires social dominance to optimize transmission of own genes. However, in SO these needs also cause substantial centrifugal tendencies within their social system.

On the other hand, SO aim at survival, a long existence preferably counted in millenia, and compete with other SO by assimilating them either by organized warfare or other forms of acquisition (Cioffi-Revilla, 1996). Their other characteristic is the establishment of social stratification with commanding elements, and a large body of elements providing food or fighting power (Gilman et al., 1981; van Leeuwen and Maas, 2010).

All this requires long-lasting social stabilization and with that subtle genetic and epigenetic adaptations in the human brains forming an SO. Hence, the cultural environment became a selective force in shaping individual brain connectomes and behavior for optimal integration into different cultures. Since culture and its transgenerational existence in traditions lastly depends on human brains and their motor output, this section discusses the genetic and epigenetic key setscrews during brain development resulting in the observed stratification of elites versus common people and the appropriate integration of the latter into various SO.

From the viewpoint of neurosociology, there are at least three options to optimize long term stability of ancient and modern societies, namely by shifting neuronal networks in favor of the verbal output system, by infantilizing the adult brain, and finally by moderately reducing the population IQ by shifting the Bell curve (Herrnstein and Murray, 1994) a little to the left. But how?

### Increasing the language network at the expense of executive abilities

Evolutionarily, a major socially stabilizing factor was shifting the control of motor output during intragroup aggression away from the visuomotor network using physical aggression to the verbal homunculus network handling non-physical output with verbal and non-verbal components (Progovac and Benítez-Burraco, 2019). On the receptive side is the penchant to obey and follow verbal orders. Both audiomotor output and input orchestration requires more audition-related cortex and computational space provided by unimodal association cortex. Thus, functional dominance of the language network might be achieved by increasing the portion of the verbal homunculus and/or by impaired switching between the visuomotor and audiomotor networks. Given the relatively easy genetic or developmental re-routing of thalamocortical and intracortical connections, one would also expect corresponding changes in the connectome limiting diverse higher executive functions such as completing many discrete tasks in particular time slots, dealing with unexpected outcomes, performing tasks with different motor characteristics, and tasks with no immediate feedback on success (Chan et al., 2008), while language-related networks are poorly prepared for this as explained in section “5.1 Functional limitations of language-related thinking.”

### Infantilization of adult brains: neoteny and prodigies

Neoteny and self-domestication of the human species as evolutionary trends are widely accepted notions (Benítez-Burraco et al., 2018; Bednarik, 2023). Thus, it appears reasonable to expect that the underlying genetic and developmental processes could regulate individually the retention of juvenile traits into adult life. Setting aside here the genetic or neurobiological causes of these phases, e.g., myelination, the focus is on the developmental time point as this is critical for the theory postulating limited cognitive and executive abilities in adulthood.

The end of classic human childhood has been defined to occur after 7–8 years, with a tendency to shortening compatible with an evolutionary trend (Paquette and Bigras, 2018). There appear to be no distinct molecular markers for the end of childhood; proxies such as telomere length, DNA methylation age and various other biological markers did not correlate well with chronological age (Robinson et al., 2023). Likewise, developmental fMRI studies usually start with early puberty (Ramsden et al., 2011). An important timepoint occurs when children do no longer believe in fairy tales that usually link visual imagery so tightly with words that the tales become real. This seems to coincide well with the end of childhood around the age of 8 or 9 years bringing complete disbelief in seasonal fantastical figures such as Santa Claus and the Easter Bunny (Prentice et al., 1978; Blair et al., 1980; Woolley and Cornelius, 2013). Functionally, this corresponds to the distinction between magical thinking and causality in the real world (Subbotsky, 2004). Thus, subtle shifts in developmental timing of brain maturation could generate gullible individuals unable to dissociate meaning of words from reality.

Some hints supporting this view come from the study of prodigies which indicate early emergence of talents and/or high IQs. The limited opportunities for analyzing brains of prodigy children have not revealed a unitary picture (Pesenti et al., 2001; Fehr et al., 2010). Geake (2009) reviewed brain mapping studies comparing normal versus gifted children, finding some expansion and use of additional networks, mostly related to memory and increased prefrontal activation. Another factor in prodigies appears to be increased white matter density. But rather likely, the most efficient way of subtly decreasing executive abilities would be to retard the development of prefrontal cortex and associated connections.

The generally accepted theory holds that the child prodigy phenomenon is a result of an exceptionally accelerated mental development (Shavinina, 2016) but there are arguments to the contrary: normal brain development of children is a developmentally retarded process enabling social integration during an evolutionarily protracted childhood, and the occurrence of prodigy is just a developmental failure and thus rare. No human social structure, be this the small societies as during most of human evolution nor the complex societies today, could function with too many children being smarter than their parents. Therefore, normal brain maturation in children might be a process that involves plugging brain development as to prevent too rapid development leading to social instability. But this plugging process could also be useful to produce brains with suppressed cognitive potential yet stabilizing SO by creating a penchant to believing in words unrelated to reality - in other words simplemindedness.

### IQ shifting

To avoid discussing the many variants and meanings of measuring intelligence, IQ is taken here as a proxy reflecting a mixture of different talents and fast processing. For simplicity, talents may reflect optimal developmental partition of the cortex, while fast processing is usually subsumed as the G-component of intelligence (Geake, 2008, 2009; Geake and Hansen, 2010). From a neurobiological point of view, this means fast processing of internal and external signals across the brain, rapid switching between different network states (Polunina and Davydov, 2006), and fast pattern separation and completion (Kyle et al., 2015; Liu et al., 2016; Li et al., 2020; Stevenson et al., 2020; Nash et al., 2021). Transgenerational fluctuations of the mean IQ have been reported (Flynn, 1987) but were based on approximated normal distributions in different cultures and periods. Thus, in terms of behavioral genetics, the presented scenario follows largely Plomin and Deary (2015) and Plomin and von Stumm (2018) who present a balanced view of the genetics of human intelligence.

From an academic viewpoint, it appears counterintuitive to lower IQs, for usually smarter means better. But in the framework of a SO, it brings advantages. The left-shift of the Bell curve by even a few IQ points entails more simplemindedness in the population but also a reduction of individuals in the top IQ regions and therefore fewer competitors for the intelligent and ambitious individuals. Theoretically, the easiest way to lower the G-part of the mean population IQ would be to permanently downregulate the thalamo-cortical loop activity by “dimming”, possibly also by reducing the activity levels of the monoaminergic systems in the brain stem (Figure 4). The optimal candidates to achieve such regulation are tonically active inhibitory systems (Lee and Maguire, 2014) found in many brain parts. Important players are the reticular part of the substantia nigra (inhibiting brain stem motor networks), the internal pallidum (inhibiting the thalamic throughput to the cortex) and in the central amygdala maintaining a delicate balance between neuronal networks subserving reward and aversion, possibly including social behavior (Fonberg, 1972). If these tonically inhibitory brakes become overactive, for example in the pallidothalamic axons that normally subtly inhibit motor and associative throughput from cerebellar feedback loops to the cortex, Parkinson symptoms emerge such as poker face yet also beginning dementia. Or they may cause, as in the case of the default mode network shown in Figure 7, depressive phases. Conversely, loss of these brakes because of basal ganglia degeneration in Huntington’s disease, entails overshooting movements and corresponding effects in the associative cortex resulting in the Tourette syndrome with verbal tics or shouting. Given the relative paucity of such tonically inhibitory neuron populations, these would present ideal targets for dimming or brightening the associative cortex – a single mutation in a specific synaptic receptor of these inhibitory cells might be sufficient. In short, permanent dysregulation of thalamocortical inputs underlies a variety of neuropsychiatric symptoms, and dampening this activity is likely to result in suboptimal cognitive abilities and lack of drive in a large population segment, but this stabilizes large SO.

### The emergence of “common people” and “elites”

The previous sections have focused on possible genetic adaptation mechanisms from Pleistocene brains to modern humans forced to adapt to live in stratified societies characterized by a majority of people inclined to talk at the cost executive abilities, and a minority using efficiently both language and executive networks to achieve an economically or administrative dominant position.

It would seem more likely that slight lowering a population IQ is due to natural selection yet initiated by epigenetic processes. For example, McFarland et al. (2022) calculated that about half of the US population had been exposed to toxic levels from leaded gasoline in childhood and extrapolated from this an average decline of 5-7 IQ points across two generations. Likewise, the epigenetic impact of lead pollution in ancient Rome has brought an estimated decrease of 2-3 IQ points (McConnell et al., 2025). In principle, simple genetic factors could result in similar shifts, because specific yet lasting epigenetic changes in a population can serve as a template for later natural selection (Lipp, 2017). Thus, “intellectual dimming” and a language network demanding more computational space could be caused by both genetic selection and assortative mating.

To the contrary, increasing intelligence by genetic or epigenetic factors is not impossible but faces problems. One is that upregulating thalamic throughput might cause “cortical brightening”, but hyperactivity of thalamocortical channels is also known to be associated with autism and schizophrenia (see section “Thalamocortical connectivity as a key target in tuning the cortical connectome”). The second problem is that elite members need superior intelligence, executive abilities and verbal fluency, obviously a composite of low probability (Gignac, 2025). Such combinations are rare since they reflect the outcome of several random processes such as shuffling of the parental genome during meiosis and, during brain development, an optimal distribution of thalamocortical channels and their ideal tuning. Hence, the emergence of elites in large societies is not genetically predetermined but a developmentally random process with a limited number of cognitively optimal results. In ancient Egypt, the percentage of elite people and their literate helpers in the population has been estimated as 2%–5% (Baines et al., 2000; Baines et al., 2007; Driaux, 2020). Translating this to present day would mean that modern elites are formed by individuals with IQs of 120 and more, a not unlikely estimate.

In parallel and independently, the evolutionarily older multidomain neural networks supporting more demanding intellectual and/or manual executive functions appear to have been preserved in a minority of the population by non-genetic processes. A prominent factor in the spreading of intellectual and executive skills in antique societies is the migration of people escaping the rigid social structures as engineers, scientists, traders and explorers and therewith substantially promoting technical and scientific progress.

## Discussion

Having wondered that deeper thinking in the western society appears often to be determined by words rather than by facts, this paper searched for the simplest explanation in terms of genetics, brain connectivity and evolution. Obviously, postulating a newly emerging prosody and melodiousness of language as a main driver of human cultural evolution toward growing wordiness appears simplistic. However, this scenario is not unthinkable because it does not contradict traditional and contemporary findings in anatomy, brain research, anthropology, and human history, and cannot be easily refuted by singling out some debatable points. After all, vocal manipulation of many people is obvious and requires explanation. But other scenarios or variants thereof to solve the puzzle are possible. Every computer or software is tailored to deliver a specific output. For humans, this is arranged in the neocortex. Thus, the dichotomy between visuomotor and audiomotor neural networks has a physical substrate within the human homunculus and this fact merits discussion.

Section 1 dealt with aspects of human neuroanatomy often lacking in papers about brain and mind. For one, the functional potential of the old brain (up to the mesencephalon and including the basal forebrain) as compared to the neocortex is clearly underestimated, as shown most clearly by the presence of hydrocephalic humans with at least some basic cognitive capacities and motor abilities yet lacking most of their neocortex. At present, there is only one CT-documented case accompanied by psychometric assessment showing that an almost complete lack of both the cerebral hemispheres roughly corresponds to the loss of about 25 general IQ points as compared to the French population mean, and even only 17 for language tasks, all measures well in the range of normal human variation (Feuillet et al., 2007). As this paper proposes a genetic adaptation of humans to self-constructed environments in the form of megasocieties, it is obvious but beyond a detailed discussion here that genetic or epigenetic presetting of hypothalamus-based mechanisms must play an important role in this process. Specifically, this involves subtle tuning in aggression levels, impulsivity, eusociality, dominance behavior and other traits based on a regulated continuum between extreme behavioral phenotypes.

Section 2 describes in some detail the intrinsic connections of the associative cortex and limbic cortex since these also form the framework for the various modern imaging methods. The enormous somatotopic compression carrying the observable output of the human brain to the effector muscles is rarely discussed, even though it predetermines the mode of action of the associative cortex primarily devoted to preparing motor output. This analysis shows that the classic sensory inputs through the posterior thalamus are quantitatively matched, if not surpassed, by subcortical inputs through the rostral parts of the thalamus to the prefrontal association cortex. A discussion of the joint role of hypothalamic and olfactory inputs to the associative cortex inputs is not possible here, but one should note that the mammillothalamic tract (being only one of interoceptive inputs) is equal to the optic tract in terms of diameter and axon counts, about one million fibers in the optic tract. Another point is that the long intracortical rostro-caudal fibers are quantitatively less important than assumed (Rosen and Halgren, 2022). This presents a problem for traditional psychology focusing on stimulus-response processes starting in visual and auditory cortex and progressing to higher order associative cortices, including the hippocampus-projecting entorhinal cortex eventually “planning” motor output in prefrontal areas. In contrast, the motocentric view adopted here holds that the action-reaction principle is more appropriate: both mammals and humans move and adapt ongoing movement to changing interoceptive, sensory and proprioceptive impulses that are simultaneously blended in associative thalamic nuclei and predispose the associative cortical network to play on the motor cortex like an improvising harmonica player pressing the dots of his instrument.

Section 3 is an attempt to understand the functional consequences of having two very different motor neocortical output channels. It is presented by Mozart arias – after all, larynx and pharynx are the magical flute of human evolution. Neuroanatomically, this perspective contains two critical points. One is to accept that the language-related cerebellar feedback through the dentato-rubro-thalamic projection is not only targeting the motor ventrolateral thalamus but bypassing it to different anterior and dorsomedial nuclei innervating most of the associative cortex (Palesi et al., 2015). This near-simultaneous activation includes temporal, parietal, and prefrontal parts - thereby biasing the likelihood of the next emerging words or sentences. The other point is the paucity of information regarding the cortico-olivary projection with climbing fibers driving Purkinje cells with respect to language, since it makes no sense to have a large non-somatotopic modulatory system (the pontocerebellar mossy fibers) without a corresponding olivo-cerebellar input to be modified (Ausim Azizi, 2007), see also Supplementary Figure 2. Functionally, the assignment of melodiousness and prosody to the right hemisphere corresponds by and large to the current understanding of language organization. Given the massive subcortical inputs to the associative cortex, future studies on hemispheric lateralization might also search for corresponding asymmetries in hypothalamic and hippocampal structures, such as observed in studies of human fornix diameter or axon numbers found usually to be larger in the right hemisphere (Tsivilis et al., 2008; Ozdogmus et al., 2009; Senova et al., 2020).

Section 4 is more speculative by inferring different cognitive functionality and thinking modes associated with the two motor output structures. It lacks linguistic sophistication but should be considered as human ethology, that is observing the context of verbal and non-verbal human behavior (Pisanski et al., 2016). The basic idea is that one modus of thinking is visuomotor, based on the old primate inheritance of seeing, jumping, and seizing the next branch. In terms of deep thinking, this requires powerful imagination and the ability to generate, nearly parallel many different virtual pathways or patterns facilitating executive functions, be this at the managerial or the practical level. The drawback of this mode is that every imagined pattern is unique and requires an awkwardly simplified translation to wordy output. Conversely, the modus of thinking linked with the language network can be classified as audiomotor and primarily reactive. It has the power of serially associating tokens (phonemes or words) that can be combined into abstract higher order units, paving the way of understanding lastly mathematical equations (Pulvermüller, 2023). But audiomotor thinking lacks the capacity of the visuomotor system for anticipating, navigation and multiple adjustment, thus providing a short-sighted navigator at best. Perhaps it could achieve this, but to take the example of jumping from tree to tree, it would need to transform itself into a perfect echolocation system grasping the 3D-environment at once, a process requiring a long evolutionary period as in bats or whales. Thus far, the ability of the audiomotor system to use visual images seems to be limited to combining words with simple images or short visual events, e.g., as in TikTok clips. However, the audiomotor system is certainly involved in holistic assembling of sound and melodies, a topic beyond discussion here.

Section 5 is based on several theoretical papers from the author, the two examples showing that different talents and inclinations could be explained by competitive growth and subsequent pruning of thalamocortical connections during intrauterine brain development, either by inherited factors or stochastic processes – a process labeled here as developmental plasticity (Lipp, 1995; Lipp and Wolfer, 1995). One should note here that the genetic impact on brain connectivity and function varies along development. Its precision and predictability are high in the earlier periods, for example during the cerebellar migration (Rahimi-Balaei et al., 2018) and are getting looser during the formation of the associative cortex, up to the point that adult human phenotypical variability of behavior can be masked by learning, at least temporarily and under low-stress conditions. Thus, occupying initially a larger portion of temporal cortex by axons from the inferior colliculus to thalamus and cortex might entail a cascade of variably sized portions of the associative language system, at the expense the visuomotor system, or vice-versa. But once the axons are distributed, the patterns become fixed with ongoing myelination blocking axonal growth to remote places. The following intrauterine and postnatal phase include a phase of local plasticity persisting into puberty and, decreasingly, into adulthood. Cognitive variation at these stages might most likely result from modifying established thalamocortical channels, balancing their throughput by inhibition and excitation. Indeed, developmentally downregulated expression of the GluN3A NMDA receptor was identified as factor in pruning synaptic connections and presetting excitability of specific networks (Otsu et al., 2019; Bossi et al., 2023; Gonzalez-Gonzalez et al., 2023; Hurley et al., 2024) and might thus be a target for natural selection of behavioral traits.

Section 6 (from Pleistocene to Logocene) is, as most papers dealing with brain and behavior of paleolithic and neolithic humans, highly speculative in interpreting the few known facts, but the narrative is at least conceivable. The shrinking brain volume of modern humans as compared to Cro-Magnons (Balzeau et al., 2013) has apparently not been contested yet. Whether this is associated with a decrease in talents or general intelligence is debatable. However, given the lack of IQ tests dating from 35,000 years and the huge variability of modern environmental conditions, large-scale imaging studies show nonetheless a weak to moderate correlation (r about 0.25) between brain volume and psychometric measures (Betjemann et al., 2010; Lee et al., 2019; Pietschnig et al., 2022). GWA studies have identified about 1,000 genes contributing to increasing or decreasing brain size. Thus, some evolutionary decrease in intelligence related to brain size fits the presented scenario (Barber, 2010) as it seems difficult to imagine how modern “average” humans would cope with environments of the Pleistocene. Lastly, the rapid genetic adaptation to human-made environments does not fit traditional views of very slow evolutionary processes but agrees with new findings about rapid genetic adaptation in humans (Pattison, 2025).

Section 7 is likely to be the most controversial one, as it seeks the simplest way by which cognitive abilities can be regulated genetically or epigenetically during brain development and links this to stratified society structures. These include three main processes: (i) by establishing an imbalance between the manual and the language network by favoring assortative mating between verbally alike persons and thus reducing the cognitive-executive abilities associated with the visuomotor network; (ii) by protracting the developmental phase of believing in fairy tales that may account for adult gullibility, and iii) by plugging the developing brain as to prevent fast thalamocortical throughput and rapid switching between visuomotor and audiomotor networks, the loss of such developmental plugs resulting in prodigy. Therefore, genetically dependent adaptation of cognitive levels in large populations to changing environments is more likely to lower the average cognitive capacities by changing individual brain mechanisms. On the other hand, the omnipresence of elites may reflect two processes. One is the stochastic generation of individuals with optimally assembled brains during intrauterine growth, the other selection-resistant gene combinations favoring visuomotor networks, once useful in the Pleistocene yet biasing the today carriers for preferred professional activities in domains of MINT, business, trade politics and warfare.

To summarize, the novelty of the paper lies in presenting a trade-off theory between two neuronal networks shaping human culture, and the claim of prosody and melody as being an essential factor in transmitting vocally emotions and thus synchronizing human activities. The remainder is a set of better or lesser-known facts fitting the theory, some of which with an unusual perspective. From a moralistic perspective, the pervasiveness of verbal actions has certainly advanced human culture, yet at a high price by facilitating social exploitation and deadly wars. Could have human evolution taken a less deadly turn without language synchronizing emotions and actions? This would have required true telepathy, namely the wordless transmission of huge amounts of visual planning information consisting in identical and synchronized patterns of electrochemical brain activity involving two or more individuals, preferably among genetically closely related persons. This possibility appears unlikely. However, given that some Neanderthal populations showed inbreeding for 50,000 years (Slimak et al., 2024), the occurrence of such emotion-free telepathy during the Pleistocene might deserve some attention due to a recent study having re-analyzed EEG signals from an old twin study (Duane and Behrendt, 1965) by Özkurt (2024).

But can the basic idea of this paper proposing a cognitive dichotomy linked to two output systems be tested at all? Judging the heuristic value of the many mindreading studies from human brain mapping is not easy (Weder, 2022). Technically, they are conducted in magnetic tubes or seats with restrained mobility and depend much on threshold manipulations and digital masking of images. It is thus difficult to evaluate to which degree the published patterns reported for almost any psychological process reflects reality or processing artifacts. The recently emerging techniques of digital superposition from hundreds of papers (Swanson et al., 2024) may help to clarify this point, but the problem of immobility and virtual realities remains unsolved. From my own experience, EEG correlates of behavioral activities and even from navigating birds can be achieved with very small devices (Vyssotski et al., 2006, 2009; Rattenborg et al., 2016). Thus, one could expect progress of this technology as applied to humans to visualize sophisticated brain mapping (Ismail and Karwowski, 2020; Janiukstyte et al., 2023), yet by using EEG-recording helmets and portable data loggers storing daily activities based on clever sampling and analytical procedures, either in single persons or in collectives. Hopefully, this might permit to analyze how conscious thinking, yet outside the laboratory and during everyday activities (Smallwood et al., 2021; Gnanateja et al., 2022), might reflect personal imbalances of audiomotor versus visuomotor networks.

## Data Availability

The original contributions presented in this study are included in this article/Supplementary material, further inquiries can be directed to the corresponding author.

## Supplementary material

Supplementary Video 1 An excerpt from “The magic flute”, by W. A. Mozart. Source: Reproduced with permission form Unitel.

Supplementary Video 2 An excerpt from “The Marriage of Figaro” by W. A. Mozart. Source: Reproduced with permission from Unitel.
