# Supplementary material for: The cost of language: functionally over-dominant language circuits in the human brain may limit cognitive abilities and non-verbal executive functions
Source: Front Hum Neurosci. 2025 Nov 6;19:1654703. doi: 10.3389/fnhum.2025.1654703 (PMC12631762; doi:10.3389/fnhum.2025.1654703)
Supplement: Supplementary file 1 [file Data_Sheet_1.pdf]

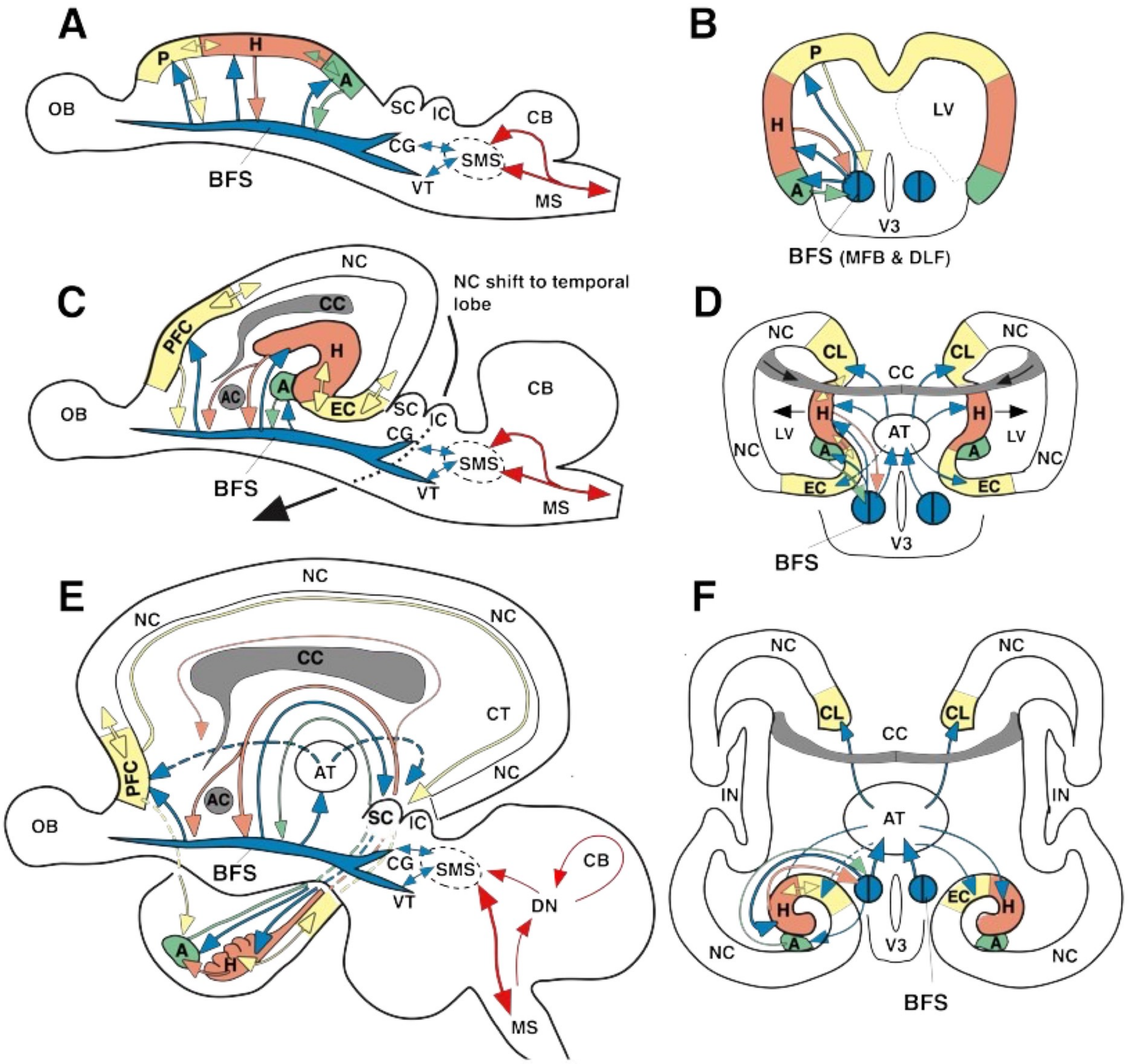

Suppl. Fig. 1. Simplified "evo-devo" sketch of the evolution of the fronto-limbic system in mammals and its connectivity with the basal forebrain system (BFS). The latter is formed by the rostral nuclei of the basal forebrain and the hypothalamic area, which are interconnected by the medial forebrain bundle (MFB, handling positive emotions and actions), with lower tail to the ventral mesencephalic area (VTA), and the dorsal longitudinal fascicle (DLF, handling chiefly negative emotions and avoidance actions), with the upper tail in the central gray region (CG). The latter structures connect to the supraspinal motors system (SMS) controlling the descending spinal motor system and its sensory feedbacks (but also priming upstream the neocortex). **A)** Lateral view of the evolutionary oldest arrangement in which the BFS establishes feedback loops with the overlaying archicortex (archipallium) for improving analysis of sensory inputs. The most rostral area (P) is analyzing olfactory inputs, becoming the prefrontal cortex (PFC), while a caudal area evolves for handling chemosensory and auditory inputs (becoming the later amygdaloid complex, A). An intermediate portion (H) becoming later the hippocampal formation analyzes activating inputs from the BSF and sends back facilitating or inhibitory feedback after having integrated inputs from both BFS and rostral and caudal archicortex. Note that the archicortex was stretched longitudinally here for better presentation of structures. **B)** Cross-sectional (coronal view) of the primordial archicortex and the newly forming lateral ventricle (LV). V3 denotes the phylogenetically old third ventricle. The blue symbol for the BFS is divided indicating its composite nature. **C)** Lateral view of a rodent-type brain.

The BSF and its caudal connections to the SMS remain unchanged, also its ascending tracts to the limbic cortex (prefrontal cortex, PFC and entorhinal cortex, EC). However, the in-growth of the corpus callosum (CC) and the development of the neocortex (NC) push hippocampal formation, prefrontal and entorhinal cortex to the rim of the hemispheres (lat. "limbus"). The anterior commissure (AC) splits the hippocampal feedback into a pre-commissural and post-commissural fornix. **D)** Cross-sectional view of the developing hemispheres shows the hippocampal formation bulging into the lateral ventricles, and the emergence of a mayor hub for the limbic cortex, the anterior (limbic) thalamus (AT) receiving unidirectional input from the BFS (through the mammillothalamic tract, MTM) thus amplifying its impact on the fronto-limbic system. **E)** Lateral scheme of the human fronto-limbic system. The BFS and its ascending activating fiber tracts and their recurrent feedbacks input largely remain, including the hub function of the anterior thalamus (Coenen et al., 2012). The shift of amygdala and hippocampal formation into the depth of the temporal lobe results in elongation of the fornix (hypothalamo-hippocampal loops) and some hypothalamo-amygdalar loops such as the stria terminalis. Entorhinal and prefrontal cortex remain connected through the cingular tract (CT) in the medial wall of the hemispheres. **F)** Cross-sectional scheme of the human brain emphasizing the hub role of the anterior thalamus. For another view of human limbic circuitry see Fig. 2. Further abbreviations: CB, cerebellum, CL, cingular cortex. DN, deep cerebellar nuclei; IC, inferior colliculus; IN, insular cortex; OB, olfactory bulb, SC, superior colliculus. Copied and modified under Creative Commons Attribution License 4.0. from Lipp and Wolfer (2022).
