# Supplementary material for: The cost of language: functionally over-dominant language circuits in the human brain may limit cognitive abilities and non-verbal executive functions
Source: Front Hum Neurosci. 2025 Nov 6;19:1654703. doi: 10.3389/fnhum.2025.1654703 (PMC12631762; doi:10.3389/fnhum.2025.1654703)
Supplement: Supplementary file 2 [file Data_Sheet_2.pdf]

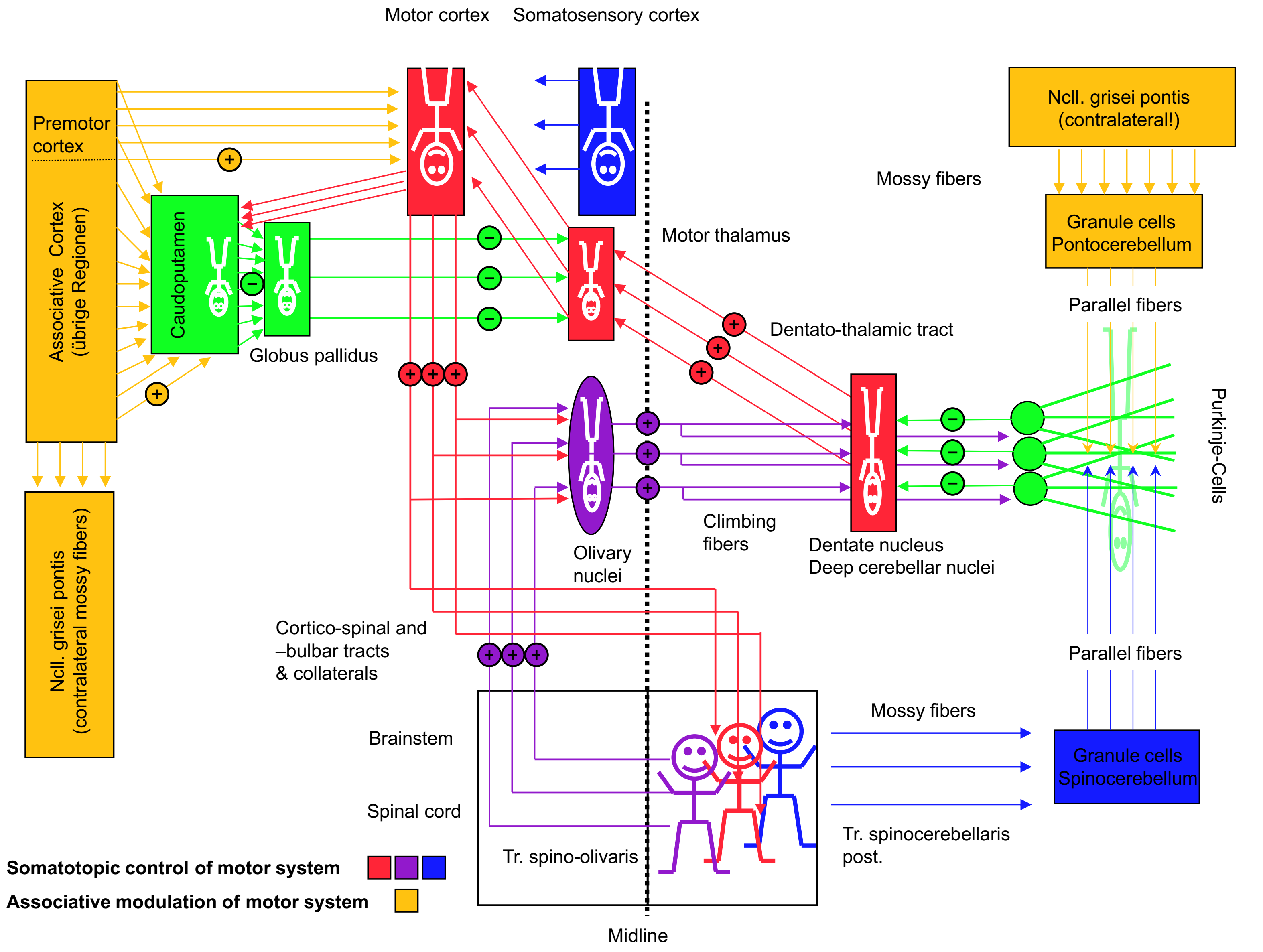

Supplemental Figure S2. Scheme of somatotopic projections between neocortex, basal ganglia, cerebellum and thalamus. Note that most projections from the associative cortex areas to the nuclei grisei pontis are not somatotopically organized. Likewise, many associative cortex regions project to the basal ganglia which in turn regulate thalamic throughput. Source: anatomy lectures H.-P. Lipp (2012)
